# Cardiac magnetic resonance imaging in the assessment of cardiac injury and toxicity from cancer-related chemotherapy

**DOI:** 10.1007/s00247-025-06260-z

**Published:** 2025-06-09

**Authors:** Jason N. Johnson, Tiffany L. Berthod, Xiaoying Cai, M. Jay Campbell, Anudeep K. Dodeja, Chris G. Goode, Kan N. Hor, Hannah M. Jacobs, Simon Lee, Yue-Hin Loke, Raymond P. Lorenzoni, Cara E. Morin, Andrada R. Popescu, Jonathan H. Soslow, Erin R. Trask, Olga H. Toro-Salazar

**Affiliations:** 1 The University of Tennessee Health Science Center, Memphis, TN USA; 2 Department of Diagnostic Imaging, St. Jude Children’s Research Hospital, Memphis, TN USA; 3 Division of Pediatric Cardiology, Connecticut Children’s, 282 Washington Street, Hartford, CT 06106 USA; 4 Siemens Medical Solution USA Inc., New York, NY USA; 5 Division of Pediatric Cardiology, Duke University Medical Center, Durham, NC USA; 6 University of Connecticut School of Medicine, Farmington, CT USA; 7 Division of Pediatric Cardiology, Nationwide Children’s Hospital, Columbus, OH USA; 8 Northwestern University Feinberg School of Medicine, Chicago, IL USA; 9 Division of Pediatric Cardiology, Children’s National Hospital, Washington, D.C. USA; 10 Department of Radiology, Cincinnati Children’s Hospital Medical Center, Cincinnati, OH USA; 11 University of Cincinnati College of Medicine, Cincinnati, OH USA; 12 Division of Pediatric Cardiology, Monroe Carell Jr Children’s Hospital at Vanderbilt, Nashville, TN USA; 13 Radiology, Connecticut Children’s, Hartford, CT USA; 14 Heart Center, Ann & Robert H. Lurie Children’s Hospital of Chicago, Chicago, IL USA; 15 Division of Pediatric Cardiology and Radiology, Le Bonheur Children’s Hospital, Memphis, TN USA; 16 Department of Medical Imaging, Ann & Robert H. Lurie Children’s Hospital of Chicago, Chicago, IL USA; 17 The Ohio State University, Columbus, OH USA

**Keywords:** Cancer survivors, Cardiotoxicity, Child, Magnetic resonance imaging

## Abstract

A dedicated guideline to the evaluation of cardiovascular toxicity in pediatric cancer by cardiac magnetic resonance imaging (MRI) does not exist. Therefore, the aim of this document is to provide guidance in the utility, acquisition, and interpretation of cardiac MRI in childhood cancer survivors. We will focus on briefly defining cardiovascular toxicity in childhood cancer survivors and reviewing the key aspects of a detailed cardiac MRI study in a patient with acute versus chronic cardiovascular toxicity. We intend for this document to serve as a guideline on how to perform a cardiac MRI to evaluate cardiac toxicity based on a review of the current literature. There will be a review of a detailed cardiac MRI protocol (bi-ventricular volumes and mass, myocardial strain, tissue characterization, late gadolinium enhancement, and 4 Dimensional/4D flow) in the assessment of cardiovascular toxicity from cancer therapy. It is hoped that the availability of a standardized approach to cardiac MRI assessment in childhood cancer survivors improves cardiovascular care in this population.

**Graphical Abstract:**

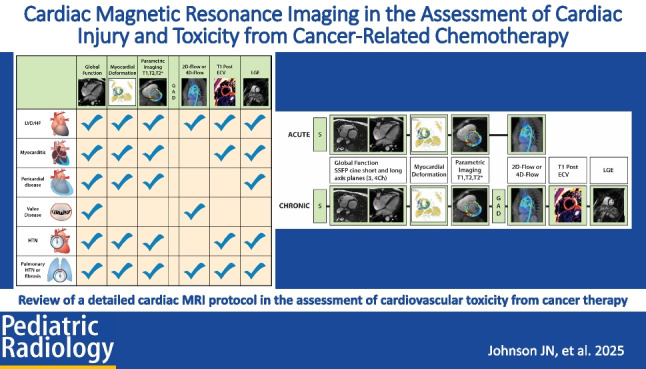

## Introduction

Cardio-oncology is an emerging discipline focused predominantly on the detection and management of cancer treatment-induced cardiovascular dysfunction (cardiotoxicity) [1, 2]. The incidence of pediatric cancer increased by an average of 0.5% per year from 2003 to 2019, and it remains the leading cause of disease-related death in US children after infancy [3]. However, 5-year survival now exceeds 85%, allowing many childhood cancer survivors to live for decades [4, 5]. As childhood cancer survivors age, cardiovascular disease becomes the leading cause of non-cancer mortality, and reducing cardiovascular risk can improve outcomes [6]. Early identification of childhood cancer survivors at risk for cardiovascular disease is critical to enhancing long-term survival.

Multimodality imaging guidelines for assessing cardiac function during and after cancer therapy are available for children and adults [7–9]. However, no dedicated guideline exists for evaluating cardiotoxicity in childhood cancer survivors using cardiac magnetic resonance imaging (MRI) as current pediatric cardiac MRI guidelines focus on the cardiomyopathy type rather than causative agents [10]. Optimal care requires collaboration among pediatric and adult cardiologists and other medical specialists. Given the limited data on cardiac MRI’s role in cardiotoxicity outcomes, this document does not provide recommendations on its impact on cancer treatment; instead, it defines cardiotoxicity in pediatric cancer patients, outlines key elements of a cardiac MRI study for acute and chronic cardiotoxicity, and serves as a guideline for performing cardiac MRI-based cardiac toxicity assessments based on current literature.

## Cardiotoxicity of cancer therapeutic agents (Table 1)

**Table 1:** Cardiotoxicity of cancer therapeutic agents

Cardiotoxic drug classification	Examples of cardiotoxic agents	Examples of pediatric cancers exposed to cardiotoxic drug classification	Cardiotoxicity as evaluated by cardiac MRI
Conventional chemotherapies			
Anthracyclines	Doxorubicin, daunorubicin, epirubicin, idarubicin, mitoxantrone	Ewing sarcoma, hepatoblastoma, leukemias, neuroblastoma, lymphomas, non-rhabdomyosarcoma, osteosarcoma, Wilms tumor	Cardiomyopathy, myocardial fibrosis, pericardial disease
Platinum-based therapies	Cisplatin, carboplatin	Hepatoblastoma, Hodgkin’s lymphoma, neuroblastoma, osteosarcoma, Wilms tumor	Arterial thrombosis, cardiomyopathy, myocardial fibrosis, systemic and pulmonary hypertension
Alkylating agents	Cyclophosphamide, ifosfamide	Ewing sarcoma, leukemias, neuroblastoma, lymphomas, non-rhabdomyosarcoma, osteosarcoma, rhabdomyosarcoma, Wilms tumor	Arterial thrombosis, cardiomyopathy, pericardial disease, pulmonary hypertension, pulmonary fibrosis, intramural changes in small coronary vessels
Antimetabolites	5-Fluorouricil, cytarabine	Ewing sarcoma, leukemias, non-Hodgkin lymphoma	Arterial thrombosis, pericardial disease, atherosclerosis, coronary spasm, myocardial ischemia
Target Molecular Therapies			
VEGF inhibitors	Bevacizumab, sorafenib	Brain tumors, hepatocellular carcinoma, leukemias	Arterial thrombosis, hypertension, cardiomyopathy, coronary artery disease
Proteasome inhibitors	Bortezomib	Leukemias, lymphomas, solid tumors	Arterial thrombosis, left ventricular dysfunction, heart failure, systemic hypertension, pulmonary hypertension, pulmonary fibrosis
mTOR inhibitors	Everolimus, sirolimus, temsirolimus	Brain tumors, rhabdomyosarcoma, solid tumors	Cardiomyopathy, atherosclerosis, coronary spasm, systemic hypertension
BCR-ABL1 inhibitors	Dasatinib	Leukemias	Pulmonary hypertension, arterial vascular disease, venous thrombus embolism
BRAF inhibitors	Dabrafenib	Brain tumors	Cardiomyopathy
MEK inhibitors	Trametinib, selumetinib, mirdametinib	Brain tumors	Cardiomyopathy, pulmonary hypertension
Immunotherapies			
CAR T-cell therapy		Leukemias, lymphomas	Cardiomyopathy, arterial vascular disease, pulmonary hypertension, pulmonary fibrosis, pericardial disease, venous thrombus embolism
Immunotherapy agents	Blinatumomab, dinutuximab, brentuximab, nivolumab, pembrolizumab, rituximab	Leukemias, lymphomas, neuroblastoma	Myocarditis, pericardial disease, acute coronary syndrome, and vasculitis
Radiation			
Radiation therapy		Lymphomas, solid tumors	Cardiomyopathy, arterial vascular disease, pulmonary hypertension, pulmonary fibrosis, pericardial disease, valvular disease, myocardial ischemia

*BCR-ABL1*, breakpoint cluster region-Abelson murine leukemia 1 gene; *BRAF*, B-Raf proto-oncogene, serine/threonine kinase; *CAR*, chimeric antigen receptor; *MEK*, mitogen-activated extracellular signal-regulated kinase; *MRI*, magnetic resonance imaging; *mTOR*, mammalian target of rapamycin; *VEGF*, vascular endothelial growth factor

Cancer therapies have well-documented cardiovascular effects (Table 1). Anthracyclines, among the most effective chemotherapeutics, target tumor cells by disrupting topoisomerase 2ß, causing DNA breaks, oxidative stress, and myocardial cell apoptosis. Anthracycline-induced oxidative stress damages DNA, proteins, and lipids through reactive oxygen species, while histone modifications alter epigenomic and transcriptomic functions [11]. Anthracyclines preferentially accumulate in cardiomyocytes’ mitochondria, contributing to anthracycline-induced cardiomyopathy [12]. Mitochondrial DNA damage underlies long-term cardiovascular risk, and anthracyclines impair progenitor cells’ regenerative capacity.

**Fig. 1:**
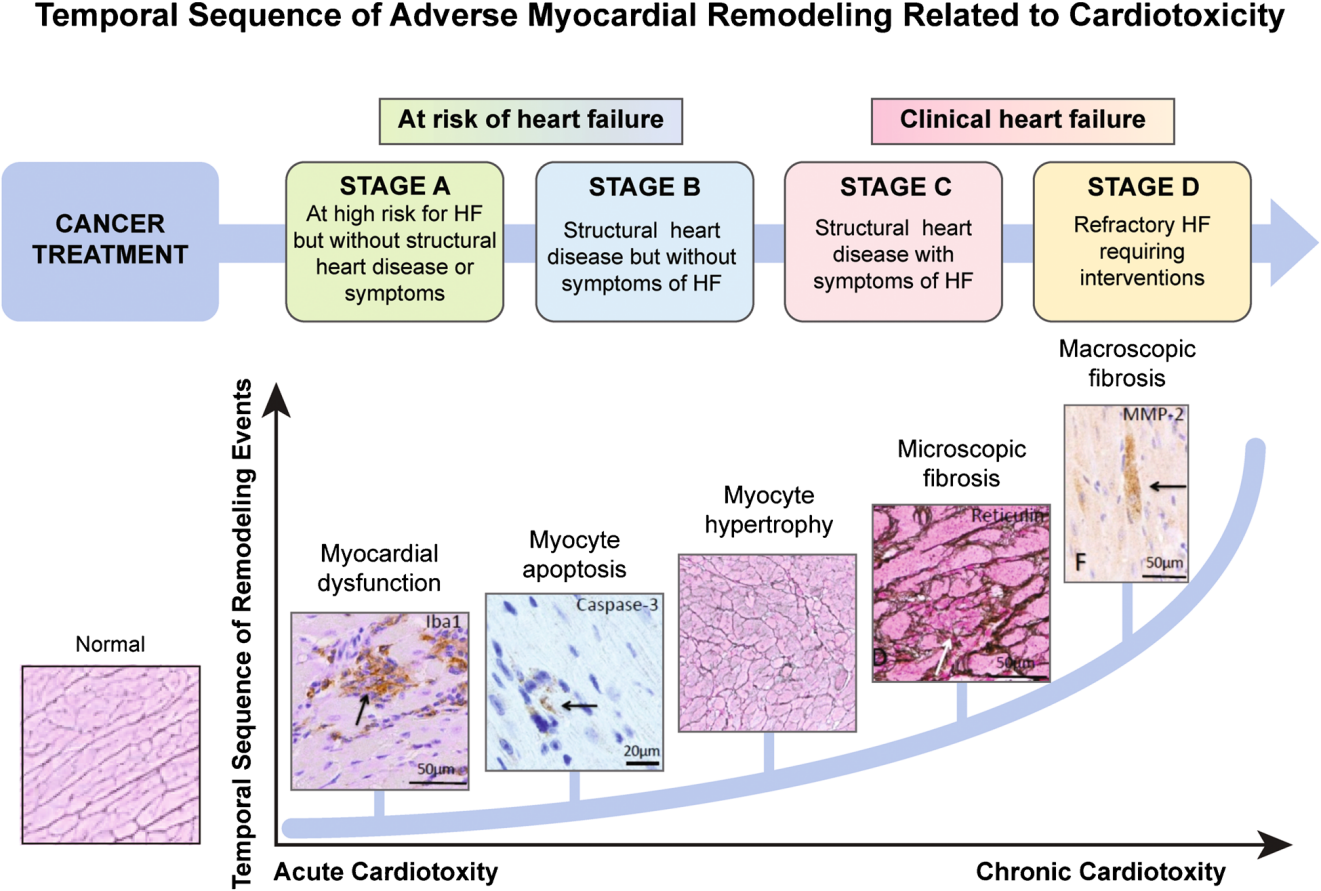
Adverse cardiac remodeling in chemotherapy induced cardiac injury. HF, heart failure

Myocyte injury has a linear relation with anthracycline cumulative dose; however, myocardial function is preserved until a critical dose or degree of damage is reached [13]. In patients treated with anthracyclines, left ventricular (LV) dysfunction begins with injury or stress on the myocardium (stage A) and may progress even in the absence of a new identifiable insult to the heart. The disease first manifests as a change in the geometry or structure of the LV (heart failure stage B). Prolonged hypertrophy and fibrosis in response to pathological signals are associated with the development of heart failure (stage C/D) (Fig. 1) [14, 15].

Targeted chemotherapy is increasingly used in pediatric cancers, improving treatment outcomes while often reducing toxicity [16]. These therapies use cancer-directed antibodies and small molecules to inhibit pathways such as platelet-derived growth factor receptor, vascular endothelial growth factor, and human epidermal growth factor receptor 2 [17]. Cardiotoxicity arises from off-target kinase inhibition, particularly affecting vascular endothelial growth factor and mitogen-activated extracellular signal-regulated kinase pathways [18]. Kinase inhibitors are linked to hypertension, thromboembolism, pulmonary hypertension, and ventricular dysfunction [19, 20].

Immunotherapies, including immune checkpoint inhibitors and chimeric antigen receptor T cell therapies, are increasingly used in childhood cancer but pose a significant cardiotoxic risk [21]. Immune checkpoint inhibitor-related cardiotoxicity, occurring in over 1% of patients, includes myocarditis, pericardial disease, arrhythmias, acute coronary syndrome, and vasculitis. Chimeric antigen receptor T cell–associated cardiotoxicity primarily results from cytokine release syndrome, causing tachycardia (mild cytokine release syndrome) to hypotension, arrhythmias, and reduced ejection fraction (EF) (severe cytokine release syndrome) [21]. Cardiotoxicity contributes to significant morbidity and mortality but can be mitigated with early immunosuppressive therapy, pretreatment evaluation, and close monitoring.

### Cardiac toxicities of radiation therapy

Thoracic radiotherapy, essential in many cancer treatments, can cause acute and chronic cardiac dysfunction [22]. Chest irradiation is linked to myocardial dysfunction, coronary artery disease, and valvular and pericardial damage. Radiation-induced oxidative stress disrupts cardiac cell function and mitochondrial integrity, increasing reactive oxygen species production [23]. Direct DNA damage and strand breaks trigger genomic instability and apoptosis. Both microvascular and macrovascular complications drive radiation-related cardiotoxicity [24].

### Cardiac MRI for monitoring pediatric cancer patients

Cardiac MRI is the gold standard for quantifying global and regional myocardial function and detecting subclinical dysfunction, including anthracycline-induced cardiomyopathy [25–28]. Variability in reported cardiotoxicity rates stems from historically imprecise definitions, addressed in the 2022 European Society of Cardiology (ESC) guidelines [29]. Early cardiac MRI markers of cardiotoxicity include low-normal global LV systolic function, increased end-systolic volume, and reduced myocardial deformation parameters, such as global circumferential strain and global longitudinal strain magnitude [30–32].

**Fig. 2:**
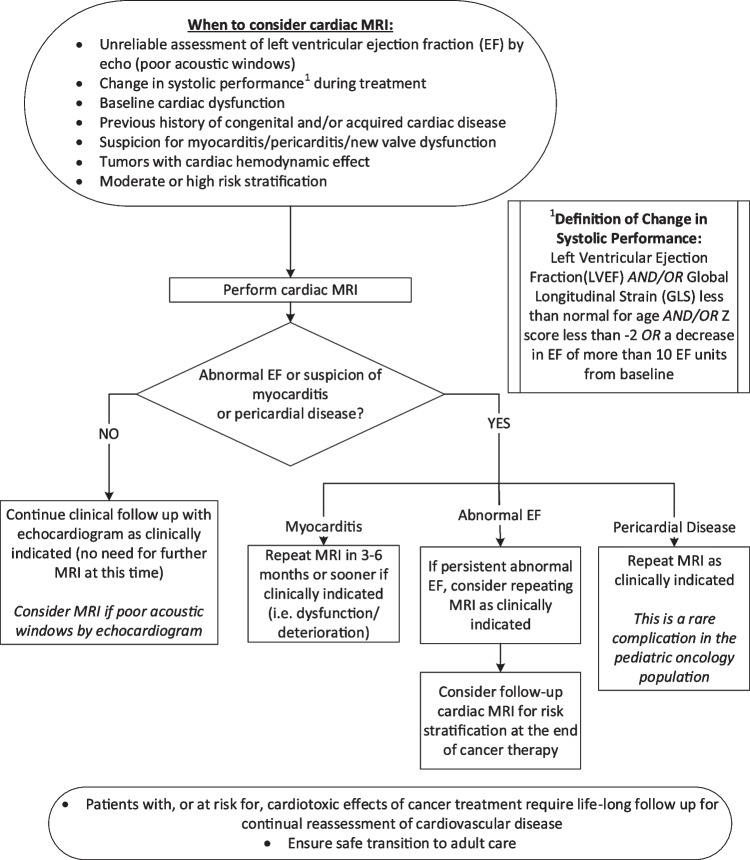
Algorithm for cardiac MRI evaluation of cardiotoxicity in pediatric cancer patients. EF, ejection fraction; GLS, global longitudinal strain; LVEF, left ventricular ejection fraction; MR, magnetic resonance; MRI, magnetic resonance imaging

**Fig. 3:**
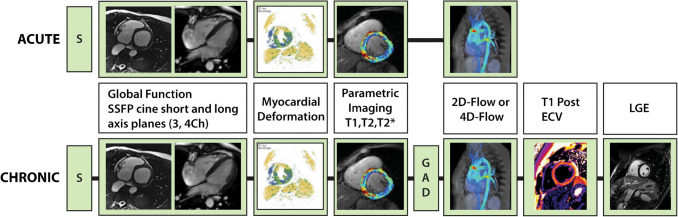
Cardiac MRI protocol for evaluation of cardiotoxicity in pediatric cancer patients. 2D, two dimensional; 4D, four dimensional; Ch, chamber; ECV, extracellular volume; GAD, gadolinium-based contrast; LGE, late gadolinium enhancement; SSFP, steady-state free precession

**Fig. 4:**
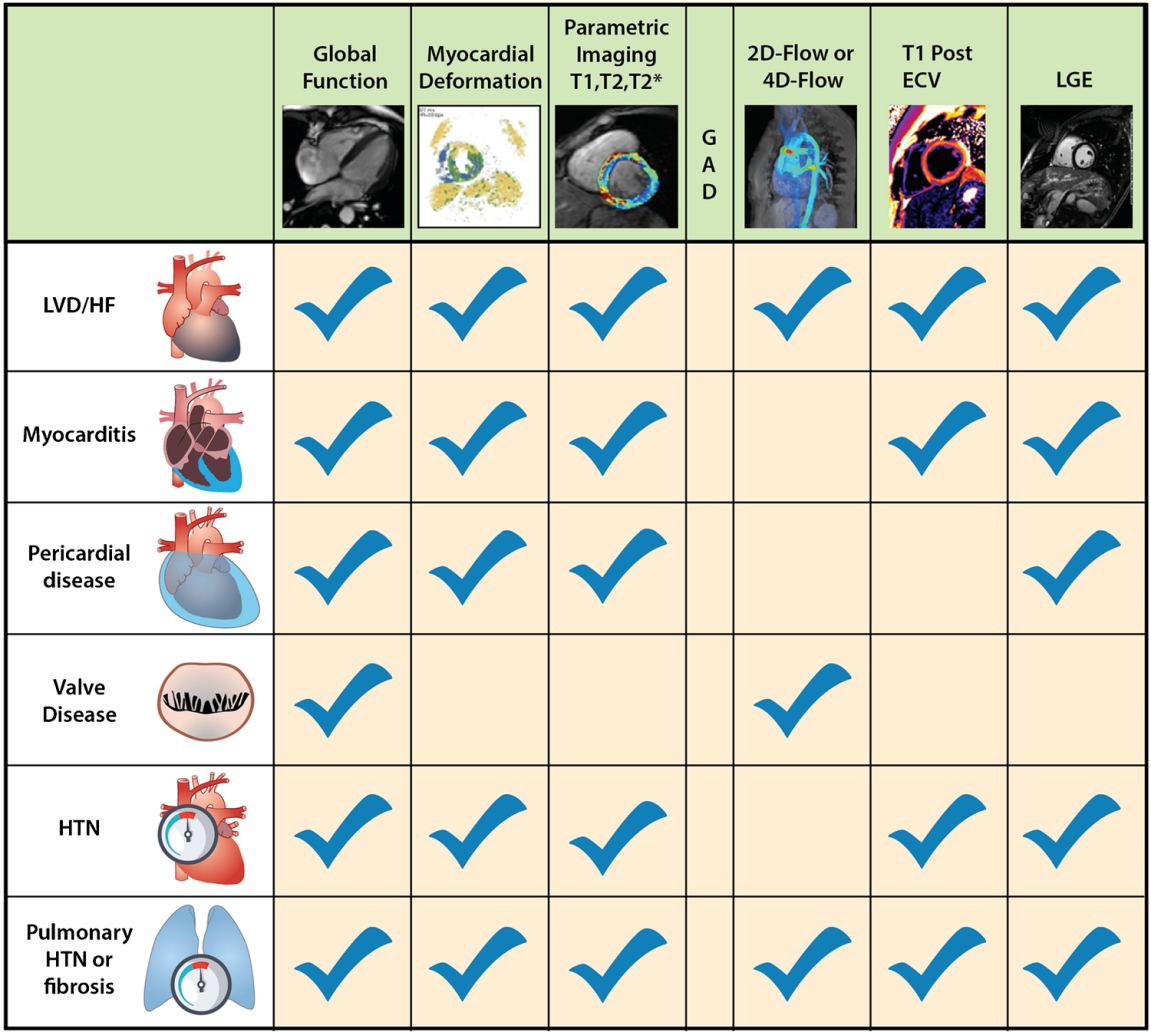
Representative cardiac MRI sequences used in evaluation of cardiotoxicity. 2D, two dimensional; 4D, four dimensional; ECV, extracellular volume; GAD, gadolinium-based contrast; LGE, late gadolinium enhancement; LVD/HF, left ventricular dysfunction/heart failure; HTN, hypertension

Suggested cardiac MRI indications for cardiotoxicity evaluation in childhood cancer survivors are outlined in Fig. 2, aligning with other proposed guidelines for cancer patients with cardiotoxic exposures [33]. This document does not cover cardiac MRI evaluation of cardiac masses as this is extensively reviewed elsewhere [32, 34]. Figure 3 presents a cardiac MRI protocol based on cardiotoxic exposure timing. In acute settings, gadolinium contrast can be omitted unless myocarditis evaluation is required, in which case the chronic protocol is recommended. Figure 4 provides a checklist of cardiac MRI sequences and their cardiotoxicity targets, with a detailed description in Table 2.

**Table 2:** Description of cardiac MRI techniques for assessment of cardiotoxicity

Clinical information	Category of cardiac MRI technique	Commonly used pulse sequences
Morphology and function		
Volumetric, mass, ejection fraction	Cine imaging	bSSFP at 1.5 T
		Spoiled gradient echo sequences (alternative choice) at 3.0 T
Myocardial deformation	Tagged imaging	Cartesian grid tags (SPAMM or C-SPAMM)
	Feature tracking	bSSFP or spoiled gradient sequences
	Strain encoding	fast Strain-Encoding (fSENC)
	Displacement encoding with stimulated echoes	DENSE
Myocardial tissue characteristics		
Inflammation	Edema imaging	T2 weighted: black blood (T2w STIR) or bright blood (T2 prepared SSFP; TSE-SSFP hybrid)
		T2 mapping: T2 prepared SSFP
Diffuse fibrosis	T1 mapping or extracellular volume fraction	Inversion recovery
		Saturation recovery, hybrid
Focal fibrosis	Late gadolinium enhancement imaging	Inversion recovery GRE or SSFP, PSIR
		Single-shot with SSFP readout
Shear stress	2D or 4D flow	2D: phase contrast imaging
		4D: GRAPPA, k-t GRAPPA, T2w-SPACE-STIR

*bSSFP*, balanced steady-state free precession; *C-SPAMM*, complementary spatial modulation of magnetization; *DENSE*, displacement encoding with stimulated echoes; *fSENC*, fast Strain-Encoding; *GRAPPA*, generalized autocalibrating partially parallel acquisition; *GRE*, gradient recalled echo; *MRI*, magnetic resonance imaging; *PSIR*, phase-sensitive inversion recovery; *SPACE*, sampling perfection with application-optimized contrast using different flip-angle evolutions; *SPAMM*, spatial modulation of magnetization; *SSFP*, steady-state free precession; *STIR*, short tau inversion recovery

### Left ventricular volume, mass, and global systolic function

Myocardial toxicity and declining left ventricular ejection fraction (LVEF) are the most recognized forms of cardiotoxicity, with cardiac imaging essential for early detection and prevention. Transthoracic echocardiography is the first-line modality due to its accessibility and ability to assess ventricular function, valvular disease, pericardial disease, and hemodynamics [35]. However, transthoracic echocardiography has key limitations, including operator dependence, interobserver variability in LVEF assessment, and geometric assumptions in calculating LV volumes, EF, and mass, particularly with 2D imaging [30]. Right ventricular (RV) size and function assessment, including right ventricular ejection fraction (RVEF), is also limited by image quality and acoustic windows. Cardiac MRI overcomes these challenges and is the reference standard for evaluating ventricular volumes and systolic function [10].

### Validation of cardiac MRI volume, mass, and global systolic function

Ventricular volume, mass, and systolic function are calculated using a cine short-axis stack from base to apex, eliminating geometric assumptions and validated in animal and human studies [36]. Cardiac MRI offers superior reproducibility with lower inter- and intra-observer and interstudy variability compared to 2D transthoracic echocardiography in heart failure patients and childhood cancer survivors [30, 37]. A recent study found that relying on 2D transthoracic echocardiography alone to detect LVEF <50% misclassified 10% of patients compared to cardiac MRI [38].

### Cardiac MRI techniques volume, mass, and global systolic function

Most cardiac MRI cine imaging uses balanced steady-state free precession (bSSFP) due to its short acquisition time and excellent blood-myocardium contrast. Ventricular analysis begins with three long-axis LV views to plan a short-axis stack from base to apex. Images are post-processed using commercial software to generate left and right endocardial and epicardial contours [39]. Reports typically include RVEF, LVEF, and ventricular mass and volumes —both absolute and indexed to body surface area for pediatric patients— such as end-diastolic and end-systolic volume, stroke volume, cardiac output, and end-diastolic mass. Cardiac MRI remains the most accurate and reproducible imaging modality for EF assessment in both children and adults.

### Regional myocardial deformation

**Fig. 5:**
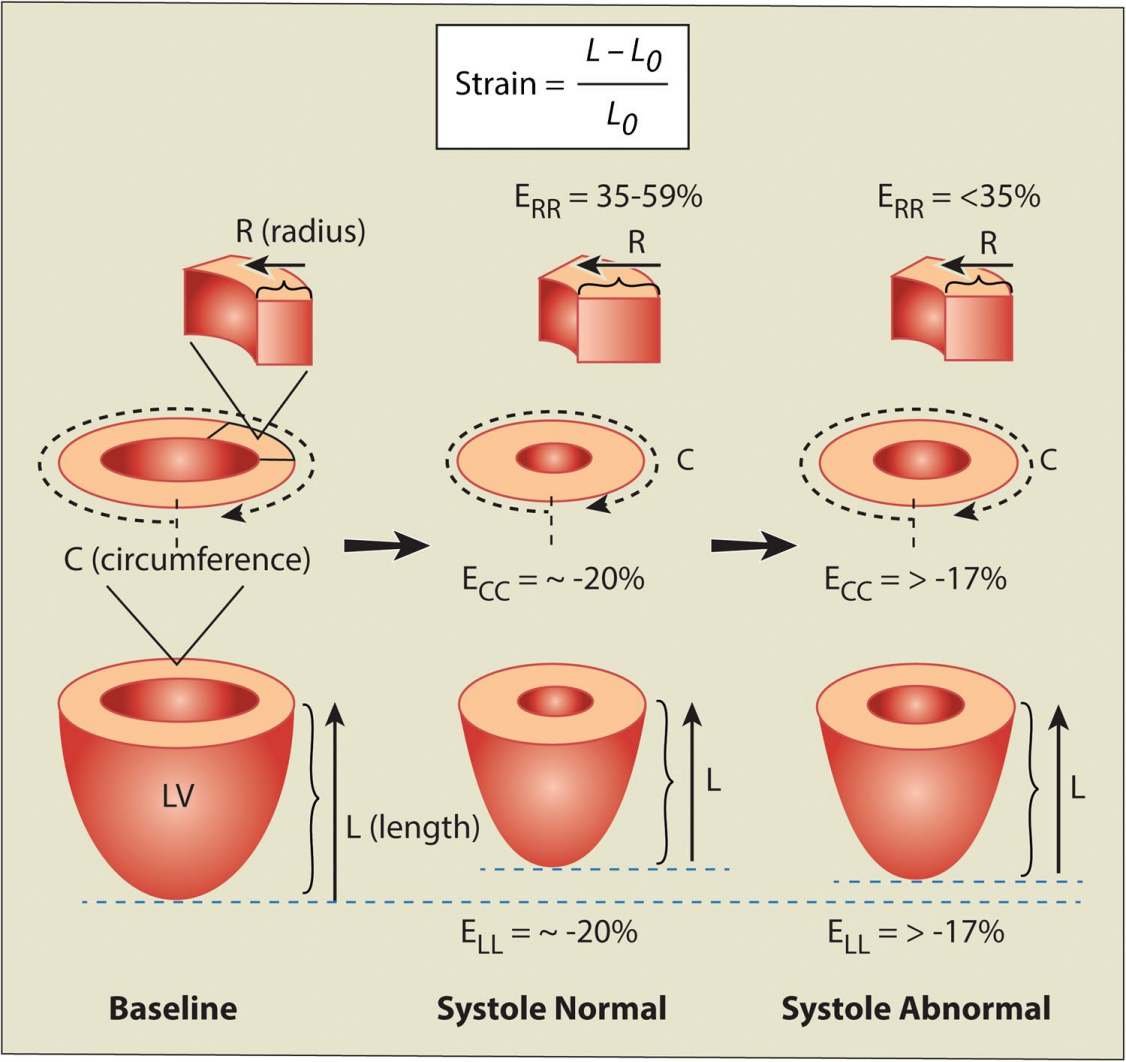
Myocardial strain. 3D circumferential-radial-longitudinal coordinate system used for strain calculation. *E*_CC_, peak global circumferential strain magnitude; *E*_LL_, peak global longitudinal strain magnitude; *E*_RR_, peak mid radial strain magnitude; *L*, current fiber length; *L*_o_, original fiber length before deformation

Strain measurements reflect changes in fiber length in a given direction and are denoted by the formula (Strain=*L* - Lo/Lo) with the value reported as a percentage. Myocardial strain is analyzed in three directions (Fig. 5). Radial strain, which measures myocardial thickening towards the ventricular center, is highly variable and not routinely used. Circumferential strain quantifies shortening around the heart’s circumference, while longitudinal strain measures base-to-apex shortening along the long axis. A decline in the strain magnitude signals myocardial weakening, with global longitudinal strain being the most sensitive marker of early subclinical myocardial dysfunction in childhood cancer survivors [40–42]. Global strain is calculated as the average of LV segmental strain values, with a less negative strain indicating reduced contractility. A strain magnitude reduction to >−17% and/or a reduction in absolute strain by greater than 15% from pretreatment baseline is of clinical significance for cardiotoxicity [43].

Strain abnormalities often precede EF decline, indicating subclinical dysfunction despite preserved global function [44]. Global longitudinal strain measured via transthoracic echocardiography or cardiac MRI predicts mortality in ischemic and non-ischemic cardiomyopathy and in anthracycline-treated patients [32, 40, 44, 45]. Moreover, *Thavendiranathan* et al. demonstrated that cardioprotective therapy guided by global longitudinal strain significantly reduced cardiotoxicity [46]. Thus, integrating strain into clinical practice could shift the treatment approach from reactive EF-based management to proactive intervention based on strain reduction.

Assessing RV myocardial strain is challenging due to its thin wall (<5 mm in healthy individuals). However, fast-strain encoding has demonstrated increasing circumferential strain from the base to the apex of the RV free wall and has demonstrated excellent inter- and intra-observer variability in healthy subjects using 3.0-T cardiac MRI [47].

Multiple cardiac MRI-based methods have emerged for the assessment of myocardial deformation, including feature tracking analysis, tagging, fast-strain encoding (fSENC), and displacement encoding with stimulated echoes (DENSE) [48–50].

### Tagged cine images

Cardiac MRI myocardial tagged imaging allows visualization of myocardial deformation without implanting physical markers and is considered the gold standard [48]. Harmonic phase strain (HARP) analysis of cardiac MRI tagged images is the preferred method for assessing the heart’s multidimensional strain direction [50, 51]. Usually, three sets of short axis images (covering basal, mid, and apical locations) and one set of four-chamber cine grid tagged images are acquired for evaluating LV myocardial contractility. Occult cardiac dysfunction with abnormal strain magnitude in both early and late cardiotoxicity in the setting of preserved EF has been reported [27, 40, 52, 53].

### Strain analysis by feature tracking

Feature tracking analysis estimates myocardial strain from standard bSSFP cine images without need for additional acquisitions. Using optical flow techniques, feature tracking analysis tracks myocardial deformation across the cardiac cycle and has been validated against harmonic phase strain analysis cardiac MRI tagged analysis [54–57]. Feature tracking analysis-derived strain has been shown to be superior to conventional functional cardiac MRI parameters, reinforcing its role in risk stratification [58]. Furthermore, mid-ventricular circumferential strain by feature tracking analysis is a reliable marker of myocardial deformation in childhood cancer survivors with anthracycline-induced cardiomyopathy [41, 59].

### Strain-encoded imaging

Strain-encoded imaging was introduced in 2001 as a method of measuring longitudinal strain in a short-axis section of the heart based on harmonic phase analysis [60]. Advances in the pulse sequences enabled single heartbeat acquisition (fast-strain encoding) significantly reducing scan time [61]. Extensively validated in in vitro and in healthy subjects, fast-strain encoding imaging closely correlates with cardiac MRI-tagging. Unlike conventional tagging, fast-strain encoding applies an out-of-plane phase-encoding gradient orthogonal to the image plane [62]. Fast-strain encoding (fSENC) measures longitudinal strain from short-axis images (basal, mid, and apical) and circumferential strain from long-axis images (four-chamber, two-chamber, and three-chamber).

Fast-strain encoding (fSENC) quantifies global longitudinal strain and global circumferential strain, along with regional systolic and diastolic dyssynchrony metrics such as the standard deviation of time to peak strain, correlation (consistent timing and shape of longitudinal strain curves), and pre- and post-systolic septal slopes, adding to our understanding of myocardial mechanical adaptation to cardiac injury [63, 64].

**Fig. 6:**
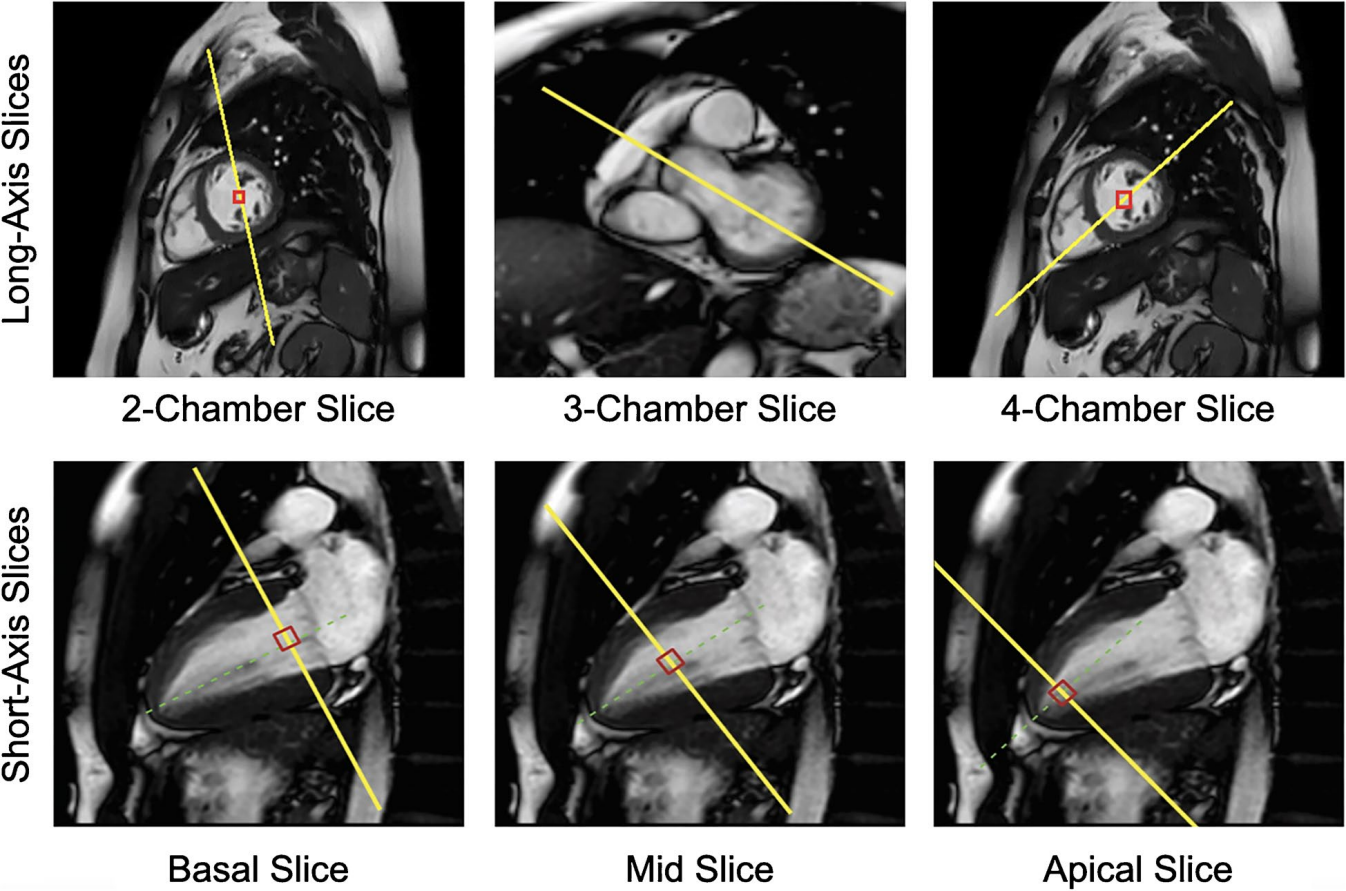
Fast-strain encoded (f-SENC) cardiac MRI protocol prescriptions. MyoHealth prescription in a 14-year-old male with Ewing sarcoma and anthracycline-induced cardiomyopathy. Six pre-contrast, separate fast-strain encoding acquisitions of the LV were performed at end systole. One of each: 2-chamber, 3-chamber, and 4-chamber slices, and three separate single short-axis slices were obtained at the base, mid, and apical levels. 2-Chamber: Prescribed off 4-chamber cine and mid short-axis stack. 3-Chamber: Prescribed off 2-chamber and basal short-axis cine. 4-Chamber: Prescribed off 2-chamber and a mid-short axis stack at end systole

**Fig. 7:**
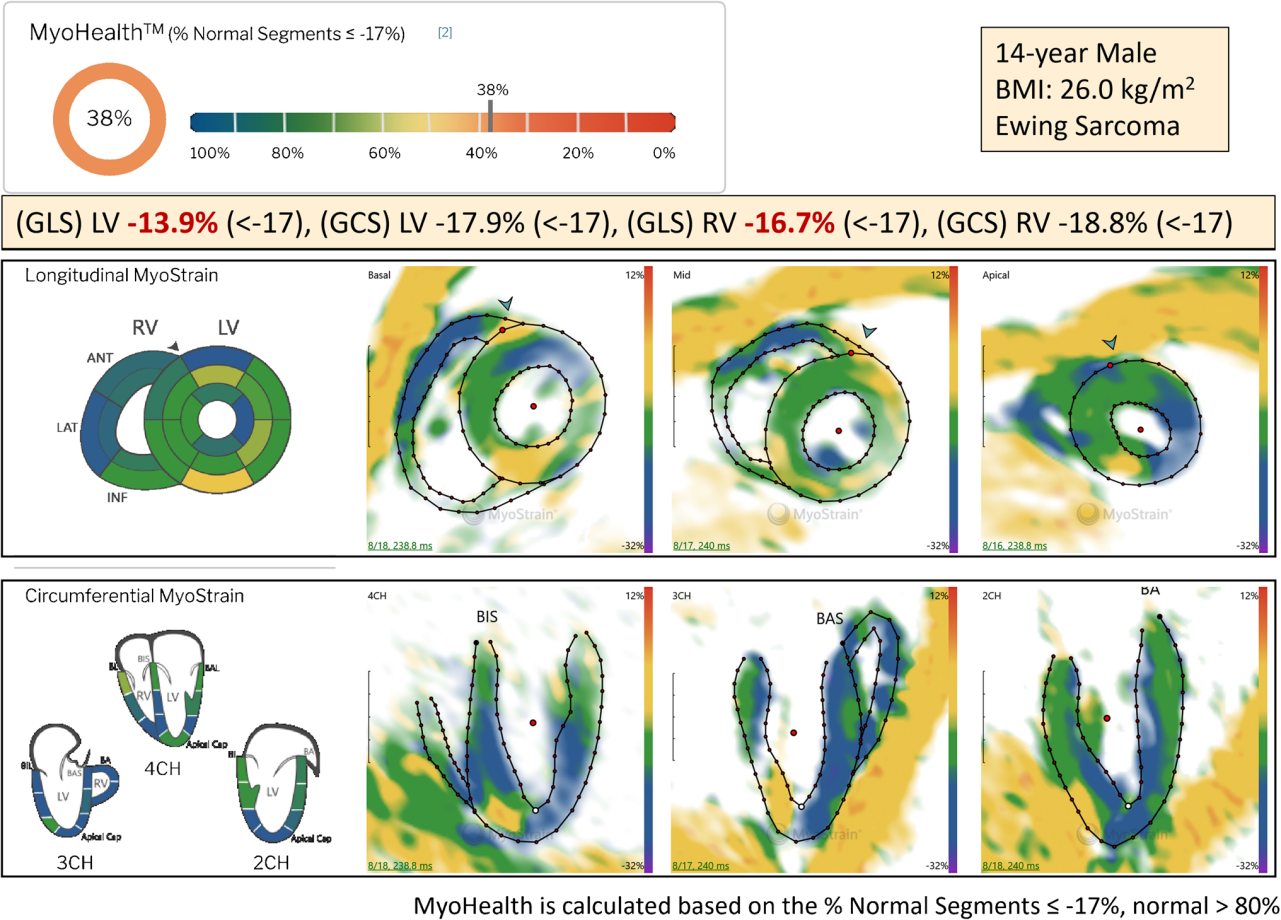
MyoHealth calculation in a 14-year-old male with Ewing sarcoma and anthracycline-induced cardiomyopathy. MyoHealth score calculation using the software provided by Myocardial Solutions Inc., Morrisville, NC, USA. The software automatically delineates the myocardium, providing a color-coded illustration of contracting myocardial tissue throughout the cardiac cycle. 16 segments from the basal, mid, and apical short axis views were used for the assessment of global longitudinal strain, and 21 segments from the two-, three-, and four-chamber views were used for the assessment of global circumferential strain. The same patient as Fig. 6. 2 CH, two chamber; 3 CH, three chamber; 4 CH, four chamber; BMI, body mass index; GCS, global circumferential strain; GLS, global longitudinal strain; LA, long axis; LV, left ventricle; RV, right ventricle; SA, short axis

An optimized protocol prescription is crucial for post-processing (Fig. 6). MyoStrain software (Myocardial Solutions, Inc.) generates a MyoHealth score defined as the percentage of LV segments with normal strain (≤ -17%) (Fig. 7). A score >80% differentiates healthy individuals from subclinical disease and predicts heart failure–related outcomes, including mortality, hospitalization, and new-onset medical treatment [65]. MyoHealth is a sensitive tool for early detection and prediction of cardiac dysfunction in cancer patients undergoing cardiotoxic chemotherapy [64]. Moreover, intra-myocardial fast-strain encoding is less impacted by compensatory mechanisms than transthoracic echocardiography and conventional cardiac MRI, making it a superior modality for monitoring chemotherapy-induced cardiotoxicity [66]. Figure 7 shows decreased global longitudinal strain and global circumferential strain measurements from a patient with anthracycline-induced cardiomyopathy with concomitant low normal LVEF.

### Cine displacement encoding with stimulated echoes (DENSE)

Cine displacement encoding with stimulated echoes (DENSE) is a well-established cardiac MRI strain imaging technique that encodes tissue displacement into image phase, generating pixel-wise displacement maps and myocardial strain maps [67–69]. Validated through rigorous phantom experiments and in vivo studies, it provides highly reproducible global and segmental strain measurements [70–72]. Cine displacement encoding with stimulated echoes shows promise for detecting subclinical dysfunction in conditions such as type-2 diabetes and post-chemotherapy cardiotoxicity [73–75]. Despite its potential, cine displacement encoding with stimulated echoes faces challenges, including long acquisition times and limited commercially available post-processing solutions. Ongoing development aims to address these limitations and facilitate broader adoption [76, 77].

### Strength and weakness of various srain techniques, reference values, and reproducibility of various strain techniques

Strain analysis outperforms conventional cardiac MRI techniques in detecting occult cardiac injury with preserved LVEF. However, strain values are not interchangeable across techniques, each with normal reference values. Harmonic phase strain analysis of cardiac MRI tagged images is the gold standard, offering reliability and reproducibility, but it is limited by spatial resolution, tag fading, and motion artifacts, making diastolic strain assessment challenging. Fast-strain encoding (f-SENC), using linear tagging, provides higher spatial resolution, eliminates breath-holding, and improves scanning efficiency. Displacement encoding with stimulated echoes (DENSE), offers high spatial resolution and rapid post-processing but has low temporal resolution. Cardiac MRI tagging, fast-strain encoding, and displacement encoding with stimulated echoes require prospective image acquisition, with the latter two being less widely available. Feature tracking analysis can be retrospectively applied to any bSSFP cine images. However, feature tracking analysis estimates strain by tracking fluid-tissue boundaries based on unique features or pixels, and variation in software algorithms yields differing numerical values [78, 79]. Despite the differences, all strain techniques surpass conventional cardiac MRI parameters in detecting subclinical myocardial dysfunction before overt cardiac impairment.

### Left ventricular diastolic function

Many forms of heart failure progress from diastolic dysfunction to systolic dysfunction, and cancer-related cardiotoxicity may follow a similar trajectory [80–82]. This progression offers an opportunity for early detection and intervention in at-risk cancer survivors. Combination therapy for breast cancer with trastuzumab, anthracyclines, and radiation therapy has been linked to diastolic dysfunction, with some studies showing systolic dysfunction only after diastolic impairment [83–85]. Pediatric radiation therapy patients exhibit worse tissue Doppler measures than those receiving chemotherapy alone, though clinical significance remains unclear [86]. Not all studies consistently demonstrate diastolic dysfunction following chemotherapy. A recent study reported that only 2% of adult childhood cancer survivors without systolic dysfunction exhibited diastolic dysfunction [87].

Echocardiography remains the gold standard for assessment of diastolic function, but cardiac MRI applications are becoming more widespread. Two-dimensional phase contrast imaging can be used to measure mitral valve inflow and pulmonary venous flow velocities, similar to Doppler echocardiography [88]. Four-dimensional (4D) flow can also be used to evaluate diastolic dysfunction (see discussion of 4D flow for more comprehensive assessment) [89]. Cardiac MRI provides superior measurements of atrial size and function due to improved visualization and volumetric analysis [90, 91]. Additionally, cardiac MRI provides the capability to assess ventricular filling curves, which have been shown to be valuable in evaluating multiple cardiac diseases [92]. Furthermore, strain imaging contributes to diastolic assessment by quantifying left atrial strain and strain rate, LV untwisting, and LV diastolic strain rate, offering additional insight into diastolic function [93, 94].

Despite growing research on cardiac MRI assessment of diastolic function, its application in cardio-oncology remains limited. Barbosa et al. reported worsened early diastolic longitudinal, circumferential, and radial strain rates following anthracycline exposure, with all three measures correlating with anthracycline dose and LVEF [95]. Van Schinkel et al. observed decreased E/A ratios and increased atrial peak filling rates after chemotherapy in patients with testicular cancer [96]. Song et al. demonstrated abnormalities in peak filling rate indexed to LV end-diastolic volume at 18 months post-chemotherapy and trastuzumab for breast cancer, though these were no longer significant after heart rate correction [97]. While cardiac MRI holds promise for diastolic assessment in cardio-oncology, further studies are needed before it can be integrated in clinical practice.

### Right ventricular cardiovascular effects

Radiation exposure commonly leads to RV free wall abnormalities or reduced RV systolic function due to the RV structural vulnerability within the thoracic cavity [24]. Radiation can also impair right coronary branches, causing isolated RV dysfunction and damage to the cardiac valves, leading to tricuspid (and less commonly pulmonic) stenosis or regurgitation. While modern stereotactic radiotherapy minimizes lung damage, older techniques or bilateral pulmonary treatments may induce pulmonary hypertension, chronically affecting RV function.

Although cardiac MRI cannot directly assess RV pressure, it is the most accurate imaging modality for assessing RV volumes, septal geometry, RV mass/hypertrophy, and RV function [98]. It provides high-resolution time-resolved 3D imaging distinguishing the blood pool and myocardium without relying on geometrical assumptions, overcoming the limitations of alternative imaging modalities.

Many chemotherapeutic agents that affect LV function also impact the RV [99]. Research suggests RV myocardial cells are more prone to apoptosis and heart failure progression than LV cells [100]. Although cardiotoxicity is often asymptomatic, its long-term impact is substantial [101]. Data on the independent prognostic value of RV function in cardiotoxicity are limited [102]. Rossetto et al. propose defining subclinical cardiotoxicity as a ≥10% reduction in 3D RVEF or ≥15% decline in RV free-wall strain, aligning with LV guidelines; however, this definition has yet to be validated [102]. A >12.7% decline in baseline RVEF assessed by 3D transthoracic echocardiography has been associated with an increased risk of cardiovascular adverse events [103]. Given the limitations of transthoracic echocardiography in achieving complete RV visualization, cardiac MRI may provide a more accurate and predictive assessment of RV cardiotoxicity, though further validation is required.

### Tissue characterization: T1 mapping, T2 mapping, and T2* imaging

#### Insights into how T1 and T2 mapping, and T2* imaging techniques are performed

Unlike other imaging methods, cardiac MRI uniquely evaluates the myocardium, offering a quantitative evaluation of myocardial fibrosis, edema, iron deposition, and mass [26, 104]. The Society for Cardiovascular Magnetic Resonance (SCMR) has consensus recommendations for how to perform standard parametric mapping techniques, including T1, T2, and T2* myocardial mapping [105]. Compared to qualitative methods, parametric mapping allows improved detection of diffuse disease and decreased interobserver variability [106]. Reference values for myocardial T1, T2, and T2* are available, but local reference ranges generated from datasets that were acquired, processed, and analyzed in a consistent way should be primarily used [105, 107, 108]. We advocate for two sets (children and adults) of local reference ranges for both native T1 and T2 mapping to allow for high-precision evaluation [10, 105].

### Relevance of measurements in identifying myocardial fibrosis, edema, and iron deposition

#### T1 mapping

Measuring myocardial T1 relaxation times before and after administration of gadolinium-based contrast agents allow for calculating extracellular volume fraction—a noninvasive biomarker for diffuse myocardial fibrosis that demonstrates excellent correlation with invasive histologic methods [105, 109, 110]. While qualitative changes in delayed gadolinium enhancement have long been the clinical standard to identify scars on cardiac MRI, small-magnitude diffuse fibrosis requires higher precision techniques due to the limitations of qualitative assessment in the absence of normal myocardium [105, 111]. Early clinical studies in childhood cancer survivors 2-3 years following their last dose of anthracycline showed increased extracellular volume, correlating with anthracycline dose and suggesting that extracellular volume is a useful measure of late myocardial injury [112, 113]. Adult studies have shown that increased extracellular volume is present in cancer patients exposed to high cumulative doses of anthracycline derivatives [114, 115]. An abnormality in a T1-based criterion (T1 relaxation time, extracellular volume, or late gadolinium enhancement (LGE)) is part of the revised Lake Louise criteria for the diagnosis of myocarditis and should be performed in patients exposed to immune checkpoint inhibitors [116]. Performing T1 mapping may be useful in detecting diffuse myocardial fibrosis in childhood cancer survivors.

#### T2 mapping

Myocardial T2 relaxation mapping enables detection and quantification of myocardial edema reflecting acute inflammation or injury [117]. Preclinical porcine studies suggest that anthracycline exposure causes elevation of myocardial T2 values, followed by increased extracellular volume, and a subsequent decrease in LVEF. Anthracycline-induced cardiomyopathy resulted in a rise in T2 values from 15% at baseline to 62% by 16 weeks. Reducing anthracycline exposure at the onset of T2 elevation resulted in the normalization of myocardial inflammation, prevention of fibrosis, and no decrement in LVEF [118]. However, clinical data is less definitive. In a prospective study of 136 adults with breast cancer receiving anthracycline and trastuzumab, T2 values peaked at 3 months after trastuzumab initiation, but the change was small (1.2 ms), and remained within the normal limits [119].

T2 mapping may have a growing role in immune checkpoint inhibitor-associated myocarditis. Early retrospective studies suggest T2 mapping detects myocarditis more effectively than T1 mapping and LGE [120]. Abnormal T2-based criteria, including elevated T2 relaxation times and increased signal intensity in T2-weighted imaging, are integral to the revised Lake Louise criteria for myocarditis diagnosis by cardiac MRI [116].

T2 mapping is likely most beneficial for patients at high risk of acute myocardial injury and may help to guide interventions to mitigate further damage.

#### T2* imaging

Iron overload is common in childhood cancer survivors due to frequent red blood cell transfusions in the setting of cancer-therapy-induced myelosuppression [121, 122]. Iron accumulation is toxic to many tissues and in the heart can lead to cardiomyopathy and heart failure. The degree of morbidity and mortality associated with iron overload will depend on the affected organ and the level of accumulation [123]. Guidelines recommend that patients with a history of hematopoietic stem cell transplant or those who have received multiple red blood cell transfusions should be screened with a ferritin level; those with a ferritin above 500 ng/mL should receive a T2* liver MRI to assess for liver iron content, which is a surrogate for total body iron content [124].

Measuring myocardial T2* values allows estimating myocardial iron deposition. Limited data exist on the prevalence of myocardial iron deposition in pediatric oncology patients. One group showed that liver iron overload was found in up to two-thirds of pediatric oncology patients who received multiple red blood cell transfusions at a 1-year follow-up MRI, and the degree of liver iron content correlated with the total volume of red blood cells transfused and persisted at least 2 years after the initiation of therapy. In this group, only 14% of patients exhibited myocardial iron overload as measured by T2* [125]. However, in childhood cancer survivors who have or are at high risk of anthracycline-induced cardiomyopathy, including a T2* measurement in a cardiac MRI is simple and takes less than 1 min.

### Late gadolinium enhancement in pediatric cancer patients

LGE or delayed enhancement imaging is a cardiac MRI technique used to differentiate viable from non-viable myocardium [126, 127]. This technique can be used across a wide range of myocardial disease processes, including myocardial infarction, infiltrative processes, myocarditis, and cardiomyopathies [126]. LGE is a routine component of the cardiac MRI evaluation for adult patients; however, it is less commonly used in children because of the low prevalence of myocardial disease in children, concerns about gadolinium contrast safety, and a lack of familiarity at many pediatric centers [128]. The ability to detect non-viable myocardium is a reason LGE is recommended (IIa indication) in patients with heart failure and would seem to make it an ideal tool for the evaluation of anthracycline-induced cardiomyopathy [129].

Animal models of anthracycline toxicity have noted LGE abnormalities, and these are associated with future LV dysfunction [130]. Biopsy evaluations of adult patients have also demonstrated the presence of fibrosis [131]. In adult patients with a history of anthracycline treatment evaluated with LGE, the findings are mixed. Studies report LGE findings with prevalence rates of 0-30%; however, interpreting these findings is complicated by the small sample size of these studies and inconsistent methodology [132, 133]. Recently, in a much larger study (298 patients), Modi et al noted no increase in abnormal LGE findings when compared to controls [134].

Studies of childhood cancer survivors have revealed similar results. The majority of studies evaluating this question have revealed no increased incidence of LGE abnormalities [104, 135–138]. Cheung et al. in a study of 52 childhood leukemia survivors noted 9% and 38% incidences of LV and RV fibrosis, respectively. These findings were correlated with LV and RV diastolic abnormalities [139]. Overall, it is unclear if anthracycline toxicity in pediatric cancer can be accurately detected using LGE. Future studies are needed. These studies should include larger sample sizes and standardized LGE techniques. New LGE techniques, such as black blood LGE, may increase sensitivity [140].

### 4D flow

Beyond traditional volumetrics, cardiac MRI enables advanced hemodynamic assessment through 4D flow imaging, an extension of 2D phase contrast imaging that encodes flow in all three spatial directions across the cardiac cycle [141]. In addition to qualitative flow visualization and precise quantification of valvular regurgitation, 4D flow allows for post-processing of biomechanical parameters that assess ventricular efficiency and vascular hemodynamics [142, 143].

A key advantage of 4D flow is its potential to enhance diastolic function assessment, overcoming the limitations of conventional 2D techniques (e.g., tissue Doppler imaging by transthoracic echocardiography, 2D phase contrast of mitral inflow) limited to data obtained in one direction/plane [144]. Novel flow metrics, including kinetic energy (the “work” of blood movement) and vorticity (the “spin” of blood), provide insight into abnormal filling patterns of patients in myocardial disease. For instance, disruptions in LV vortex formation may reduce kinetic energy conservation and contribute to thrombus formation [145].

Flow component analysis further refines ventricular efficiency by differentiating direct flow (blood transiting through the ventricle in a single cardiac cycle) from retained inflow or delayed ejection flow occurring over multiple cardiac cycles [142]. Alterations in these components correlate with lower cardiac reserve in cardiomyopathy, while the kinetic energy of direct flow is independently predictive of functional capacity in heart failure [145]. Additionally, intraventricular pressure gradients derived from 4D flow can be used to quantify hemodynamic forces exerted by blood flow on the myocardium, which reflect abnormal diastolic function and LV dyssynchrony [146, 147].

While direct applications of 4D in chemotherapy-related cardiac injury remain limited, its continued advancement may further improve the detection of subclinical cardiotoxicity in cancer survivors [143].

### Cardiac MRI limitations

While cardiac MRI offers superior tissue characterization and quantitative assessment, it has multiple limitations when compared to transthoracic echocardiography, primarily related to availability, portability, and patient cooperation. Transthoracic echocardiography is widely accessible and easily portable, and can be performed at the bedside, in off-site clinics, or even in resource-limited settings. Cardiac MRI is generally limited to specialized centers and requires a patient to be in a magnetic field.

Cardiac MRI examinations are typically longer in duration and often require the patient to remain still and hold their breath to optimize imaging quality; in younger children, typically under 7-8 years of age, sedation or general anesthesia can be required [10]. However, significant advancements have improved accessibility and reduced acquisition time. Innovations such as real-time imaging, free breathing sequences, parallel imaging, and compressed sensing, motion correction, and novel k-space filling techniques have enhanced feasibility, particularly for pediatric and non-cooperative patients [42].

### Definition of cardiotoxicity and screening cancer survivors

The management of patients diagnosed with cancer-therapy-related cardiotoxicity has important implications. However, there is currently no universally accepted definition of heart failure due to cardiotoxicity. Existing guidelines primarily rely on serial declines in LVEF and/or a reduction in global longitudinal strain magnitude for diagnosis [148, 149]. Transthoracic echocardiography is the first-line imaging modality for evaluating patients at risk for cardiotoxicity, including childhood cancer survivors [9]. Risk stratification for cardiotoxicity is based on cumulative exposure to anthracycline derivatives and/or radiation to the chest, categorizing survivors as low-, moderate-, or high-risk [150]. Current screening guidelines recommend transthoracic echocardiography every 2 years for patients with high-risk exposures (≥250 mg/m^2^ anthracycline, or ≥30 Gy chest radiotherapy, or ≥100 mg/m^2^ anthracycline combined with ≥15 Gy chest radiotherapy), transthoracic echocardiography every 5 years with moderate-risk exposures (100–249 mg/m^2^ anthracycline, or 15-29 Gy chest radiotherapy), and no screening transthoracic echocardiography for patients with low-risk exposures (<100 mg/m^2^ anthracycline, or <15 Gy chest radiotherapy) [150].

Cardiac MRI is not recommended as an initial screening modality but may be a reasonable alternative in situations in which transthoracic echocardiography is technically inadequate or as indicated in Fig. 2. While cardiovascular biomarkers are not currently part of routine screening, emerging data suggest that combining abnormal biomarker levels with reduced global longitudinal strain magnitude may enhance early detection of cardiotoxicity [151, 152]. A detailed review of screening strategies is beyond the scope of this manuscript.

### Future directions

Cardiac MRI plays a critical role in the diagnosis of cardiotoxicity, and ongoing advancements continue to enhance its utility in this patient population. Deep learning and artificial intelligence have improved the accuracy and efficiency of multiple cardiac MRI techniques [153–155]. Machine learning enables automatic segmentation of RV and LV contours, ensuring consistent and precise calculations of mass, volume, and function [153].

Cardiac magnetic resonance fingerprinting offers a significant advantage over current mapping techniques by simultaneously quantifying T1, T2, and proton density in a single image [156, 157]. These innovations, along with novel acquisition strategies, will reduce scan times while maintaining high-quality, reproducible data.

The role of stress cardiac MRI in detecting microvascular obstruction in childhood cancer survivors remains inconclusive [158]. However, emerging techniques such as exercise bike stress cardiac MRI have demonstrated promise in other cardiovascular diseases and may have future applications in childhood cancer survivors [159]. Blood oxygen level–dependent cardiac MRI, a contrast-free method for detecting myocardial ischemia, has yet to be studied in childhood cancer survivors [160].

Despite these advancements, challenges remain. Small pediatric oncology patient populations, variability in cardiac MRI protocols across specialized centers, and inconsistent screening practices hinder large-scale research. Multi-institutional collaborations using standardized cardiac MRI protocols and unified algorithms will be critical for advancing the field [160]. The Imaging in Cardiovascular Hematology Oncology Research (ICHOR) Consortium has been established as a pediatric multicenter collaborative dedicated to improving outcomes for childhood cancer survivors by leveraging both traditional and novel cardiac MRI parameters to enhance cardiovascular risk assessment and management.

## Conclusion

Cardiac MRI accurately and reproducibly assesses biventricular function, myocardial deformation, tissue characterization, and myocardial injury in pediatric oncology patients. The added benefits of cardiac MRI compared to other imaging modalities make cardiac MRI an advantageous tool for cardiac monitoring among oncology patients in a clinical setting. Challenges persist, including cost, efficiency, and accessibility, and must be overcome before cardiac MRI can be considered an acceptable cardiotoxicity screening modality. As new cardiac MRI techniques are constantly discovered and institutions collaborate with consistent protocols, cardiac MRI assessment will expand the knowledge of cardiovascular disease in childhood cancer survivors.

## Data Availability

No datasets were generated or analysed during the current study.

## References

[1] Brickler M, Raskin A, Ryan TD (2022) Current state of pediatric cardio-oncology: a review. Children (Basel) 9:127 35204848 10.3390/children9020127 PMC8870613

[2] Martinez HR, Beasley GS, Goldberg JF et al (2021) Pediatric cardio-oncology medicine: a new approach in cardiovascular care. Children (Basel) 8:1200 34943396 10.3390/children8121200 PMC8699848

[3] Siegel DA, King JB, Lupo PJ et al (2023) Counts, incidence rates, and trends of pediatric cancer in the United States, 2003–2019. J Natl Cancer Inst 115:1337–1354 37433078 10.1093/jnci/djad115 PMC11018256

[4] Howlader N, Noone AM, Krapcho M et al (2015) SEER cancer statistics review, 1975-2012. National Cancer Institute. Bethesda, MD. based on November 2014 SEER data submission, posted to the SEER web site. https://seer.cancer.gov/archive/csr/1975_2012/

[5] Yeh JM, Ward ZJ, Chaudhry A et al (2020) Life expectancy of adult survivors of childhood cancer over 3 decades. JAMA Oncol 6:350–357 31895405 10.1001/jamaoncol.2019.5582 PMC6990848

[6] Dixon SB, Liu Q, Chow EJ et al (2023) Specific causes of excess late mortality and association with modifiable risk factors among survivors of childhood cancer: a report from the Childhood Cancer Survivor Study cohort. Lancet 401:1447–1457 37030315 10.1016/S0140-6736(22)02471-0 PMC10149583

[7] Ehrhardt MJ, Leerink JM, Mulder RL et al (2023) Systematic review and updated recommendations for cardiomyopathy surveillance for survivors of childhood, adolescent, and young adult cancer from the International Late Effects of Childhood Cancer Guideline Harmonization Group. Lancet Oncol 24:e108–e120 37052966 10.1016/S1470-2045(23)00012-8

[8] Čelutkienė J, Pudil R, López-Fernández T et al (2020) Role of cardiovascular imaging in cancer patients receiving cardiotoxic therapies: a position statement on behalf of the Heart Failure Association (HFA), the European Association of Cardiovascular Imaging (EACVI) and the Cardio-Oncology Council of the European Society of Cardiology (ESC). Eur J Heart Fail 22:1504–1524 32621569 10.1002/ejhf.1957

[9] Mertens L, Singh G, Armenian S et al (2023) Multimodality imaging for cardiac surveillance of cancer treatment in children: recommendations From the American Society of Echocardiography. J Am Soc Echocardiogr 36:1227–1253 38043984 10.1016/j.echo.2023.09.009

[10] Dorfman AL, Geva T, Samyn MM et al (2022) SCMR expert consensus statement for cardiovascular magnetic resonance of acquired and non-structural pediatric heart disease. J Cardiovasc Magn Reson 24:44 35864534 10.1186/s12968-022-00873-1 PMC9302232

[11] Lenneman CG, Sawyer DB (2016) Cardio-oncology: an update on cardiotoxicity of cancer-related treatment. Circ Res 118:1008–1020 26987914 10.1161/CIRCRESAHA.115.303633

[12] Zhang YW, Shi J, Li YJ, Wei L (2009) Cardiomyocyte death in doxorubicin-induced cardiotoxicity. Arch Immunol Ther Exp (Warsz) 57:435–445 19866340 10.1007/s00005-009-0051-8 PMC2809808

[13] Bristow MR, Mason JW, Billingham ME, Daniels JR (1981) Dose-effect and structure-function relationships in doxorubicin cardiomyopathy. Am Heart J 102:709–718 7282516 10.1016/0002-8703(81)90096-x

[14] Cardinale D, Iacopo F, Cipolla CM (2020) Cardiotoxicity of anthracyclines. Front Cardiovasc Med 7:26 32258060 10.3389/fcvm.2020.00026 PMC7093379

[15] Ferrans VJ, Clark JR, Zhang J et al (2015) Improving the outcome for children with cancer: development of targeted new agents. CA Cancer J Clin 65:212–220 25754421 10.3322/caac.21273 PMC4629487

[16] Adamson PC (2015) Improving the outcome for children with cancer: development of targeted new agents. CA Cancer J Clin 65(3):212–220. 10.3322/caac.v65.310.3322/caac.21273 10.3322/caac.21273 PMC4629487 25754421

[17] Sullivan LA, Brekken RA (2010) The VEGF family in cancer and antibody-based strategies for their inhibition. MAbs 2:165–175 20190566 10.4161/mabs.2.2.11360 PMC2840235

[18] Herrmann J (2020) Adverse cardiac effects of cancer therapies: cardiotoxicity and arrhythmia. Nat Rev Cardiol 17:474–502 32231332 10.1038/s41569-020-0348-1 PMC8782611

[19] Jain D, Russell RR, Schwartz RG et al (2017) Cardiac complications of cancer therapy: pathophysiology, identification, prevention, treatment, and future directions. Curr Cardiol Rep 19:36 28374177 10.1007/s11886-017-0846-x

[20] Ryan TD, Nagarajan R, Godown J (2019) Pediatric cardio-oncology: development of cancer treatment-related cardiotoxicity and the therapeutic approach to affected patients. Curr Treat Options Oncol 20:56 31129800 10.1007/s11864-019-0658-x

[21] Stein-Merlob AF, Rothberg MV, Holman P, Yang EH (2021) Immunotherapy-associated cardiotoxicity of immune checkpoint inhibitors and chimeric antigen receptor T cell therapy: diagnostic and management challenges and strategies. Curr Cardiol Rep 23:11 33483873 10.1007/s11886-021-01440-3 PMC7821837

[22] Desai MY, Jellis CL, Kotecha R et al (2018) Radiation-associated cardiac disease: a practical approach to diagnosis and management. JACC Cardiovasc Imaging 11:1132–1149 30092970 10.1016/j.jcmg.2018.04.028

[23] Siaravas KC, Katsouras CS, Sioka C (2023) Radiation treatment mechanisms of cardiotoxicity: a systematic review. Int J Mol Sci 24:6272 37047245 10.3390/ijms24076272 PMC10094086

[24] Mrotzek SM, Rassaf T, Totzeck M (2020) Cardiovascular damage associated with chest irradiation. Front Cardiovasc Med 7:41 32266294 10.3389/fcvm.2020.00041 PMC7103638

[25] Shehata ML, Cheng S, Osman NF et al (2009) Myocardial tissue tagging with cardiovascular magnetic resonance. JCardiovascMagn Reson 11:55 10.1186/1532-429X-11-55 PMC2809051 20025732

[26] de Ville de Goyet M, Brichard B, Robert A et al (2015) Prospective cardiac MRI for the analysis of biventricular function in children undergoing cancer treatments. Pediatr Blood Cancer 62:867–874 10.1002/pbc.25381 25597617

[27] Grothues F, Smith GC, Moon JC et al (2002) Comparison of interstudy reproducibility of cardiovascular magnetic resonance with two-dimensional echocardiography in normal subjects and in patients with heart failure or left ventricular hypertrophy. Am J Cardiol 90:29–34 12088775 10.1016/s0002-9149(02)02381-0

[28] Ohyama Y, Volpe GJ, Lima JA (2014) Subclinical myocardial disease in heart failure detected by CMR. Curr Cardiovasc Imaging Rep 7:9269 25132911 10.1007/s12410-014-9269-x PMC4131698

[29] Lyon AR, Lopez-Fernandez T, Couch LS et al (2022) 2022 ESC Guidelines on cardio-oncology developed in collaboration with the European Hematology Association (EHA), the European Society for Therapeutic Radiology and Oncology (ESTRO) and the International Cardio-Oncology Society (IC-OS). Eur Heart J 43:4229–4361 36017568 10.1093/eurheartj/ehac244

[30] Armstrong GT, Plana JC, Zhang N et al (2012) Screening adult survivors of childhood cancer for cardiomyopathy: comparison of echocardiography and cardiac magnetic resonance imaging. J Clin Oncol 30:2876–2884 22802310 10.1200/JCO.2011.40.3584 PMC3671529

[31] Drafts BC, Twomley KM, D’Agostino R et al (2013) Low to moderate dose anthracycline-based chemotherapy is associated with early noninvasive imaging evidence of subclinical cardiovascular disease. JACC Cardiovasc Imaging 6:877–885 23643285 10.1016/j.jcmg.2012.11.017 PMC3745801

[32] Beroukhim RS, Ghelani S, Ashwath R et al (2022) Accuracy of cardiac magnetic resonance imaging in diagnosing pediatric cardiac masses: a multicenter study. JACC Cardiovasc Imaging 15:1391–1405 34419404 10.1016/j.jcmg.2021.07.010 PMC11240235

[33] Toro-Salazar OH, Ferranti J, Slavin GS, Hor KN (2016) Cardiac magnetic resonance characteristics of acute anthracycline-induced cardiotoxicity. J Cardiovasc Magnet Resonance 18:P268

[34] Morin CE, Griffin LM, Beroukhim RS et al (2023) Imaging of pediatric cardiac tumors: a COG Diagnostic Imaging Committee/SPR Oncology Committee White Paper. Pediatr Blood Cancer 70(Suppl 4):e29955 36083866 10.1002/pbc.29955 PMC10641876

[35] Baldassarre LA, Ganatra S, Lopez-Mattei J et al (2022) Advances in multimodality imaging in cardio-oncology: JACC state-of-the-art review. J Am Coll Cardiol 80:1560–1578 36229093 10.1016/j.jacc.2022.08.743

[36] Grothues F, Braun-Dullaeus R (2009) Serial assessment of ventricular morphology and function. Heart Fail Clin 5(301–314):v 19564010 10.1016/j.hfc.2009.02.007

[37] Bellenger NG, Davies LC, Francis JM et al (2000) Reduction in sample size for studies of remodeling in heart failure by the use of cardiovascular magnetic resonance. J Cardiovasc Magn Reson 2:271–278 11545126 10.3109/10976640009148691

[38] Nazir MS, Okafor J, Murphy T et al (2024) Echocardiography versus cardiac MRI for measurement of left ventricular ejection fraction in individuals with cancer and suspected cardiotoxicity. Radiol Cardiothorac Imaging 6:e230048 38206164 10.1148/ryct.230048 PMC10912891

[39] Kramer CM, Barkhausen J, Bucciarelli-Ducci C et al (2020) Standardized cardiovascular magnetic resonance imaging (CMR) protocols: 2020 update. J Cardiovasc Magn Reson 22:17 32089132 10.1186/s12968-020-00607-1 PMC7038611

[40] Toro-Salazar OH, Lee JH, Zellars KN et al (2018) Use of integrated imaging and serum biomarker profiles to identify subclinical dysfunction in pediatric cancer patients treated with anthracyclines. Cardiooncology 4:4 29900007 10.1186/s40959-018-0030-5 PMC5995570

[41] Mavinkurve-Groothuis AM, Groot-Loonen J, Marcus KA et al (2010) Myocardial strain and strain rate in monitoring subclinical heart failure in asymptomatic long-term survivors of childhood cancer. Ultrasound Med Biol 36:1783–1791 20870348 10.1016/j.ultrasmedbio.2010.08.001

[42] Contijoch F, Rasche V, Seiberlich N, Peters DC (2024) The future of CMR: all-in-one vs. real-time CMR (Part 2). J Cardiovasc Magn Reson 26:100998 38237901 10.1016/j.jocmr.2024.100998 PMC11211235

[43] Thavendiranathan P, Poulin F, Lim KD, Plana JC et al (2014) Use of myocardial strain imaging by echocardiography for the early detection of cardiotoxicity in patients during and after cancer chemotherapy: a systematic review. J Am Coll Cardiol 63:2751–2768 24703918 10.1016/j.jacc.2014.01.073

[44] Lunning MA, Kutty S, Rome ET et al (2015) Cardiac magnetic resonance imaging for the assessment of the myocardium after doxorubicin-based chemotherapy. Am J Clin Oncol 38:377–381 10.1097/COC.0b013e31829e19be 24192805

[45] Thavendiranathan P, Negishi T, Somerset E et al (2021) Strain-guided management of potentially cardiotoxic cancer therapy. J Am Coll Cardiol 77:392–401 33220426 10.1016/j.jacc.2020.11.020

[46] Korosoglou G, Giusca S, Hofmann NP et al (2019) Strain-encoded magnetic resonance: a method for the assessment of myocardial deformation. ESC Heart Fail 6:584–602 31021534 10.1002/ehf2.12442 PMC6676282

[47] Zerhouni EA, Parish DM, Rogers WJ et al (1988) Human heart: tagging with MR imaging–a method for noninvasive assessment of myocardial motion. Radiology 169:59–63 3420283 10.1148/radiology.169.1.3420283

[48] Osman NF, Kerwin WS, McVeigh ER, Prince JL (1999) Cardiac motion tracking using CINE harmonic phase (HARP) magnetic resonance imaging. Magn Reson Med 42:1048–1060 10571926 10.1002/(sici)1522-2594(199912)42:6<1048::aid-mrm9>3.0.co;2-m PMC2570035

[49] Shehata ML, Cheng S, Osman NF et al (2009) Myocardial tissue tagging with cardiovascular magnetic resonance. J Cardiovasc Magn Reson 11:55 20025732 10.1186/1532-429X-11-55 PMC2809051

[50] Garot J, Bluemke DA, Osman NF et al (2000) Fast determination of regional myocardial strain fields from tagged cardiac images using harmonic phase MRI. Circulation 101:981–988 10704164 10.1161/01.cir.101.9.981

[51] Jordan JH, Sukpraphrute B, Melendez GC et al (2017) Early myocardial strain changes during potentially cardiotoxic chemotherapy may occur as a result of reductions in left ventricular end-diastolic volume: the need to interpret left ventricular strain with volumes. Circulation 135:2575–2577 28630272 10.1161/CIRCULATIONAHA.117.027930 PMC5508602

[52] Jolly MP, Jordan JH, Melendez GC et al (2017) Automated assessments of circumferential strain from cine CMR correlate with LVEF declines in cancer patients early after receipt of cardio-toxic chemotherapy. J Cardiovasc Magn Reson 19:59 28768517 10.1186/s12968-017-0373-3 PMC5541737

[53] Bohs LN, Trahey GE (1991) A novel method for angle independent ultrasonic imaging of blood flow and tissue motion. IEEE Trans Biomed Eng 38:280–286 2066142 10.1109/10.133210

[54] Ruijsink B, Puyol-Anton E, Oksuz I et al (2020) Fully automated, quality-controlled cardiac analysis from CMR: validation and large-scale application to characterize cardiac function. JACC Cardiovasc Imaging 13:684–695 31326477 10.1016/j.jcmg.2019.05.030 PMC7060799

[55] Hor KN, Baumann R, Pedrizzetti G et al (2011) Magnetic resonance derived myocardial strain assessment using feature tracking. J Vis Exp 12:2356 10.3791/2356 PMC3074463 21372778

[56] Hor KN, Gottliebson WM, Carson C et al (2010) Comparison of magnetic resonance feature tracking for strain calculation with harmonic phase imaging analysis. JACC Cardiovasc Imaging 3:144–151 20159640 10.1016/j.jcmg.2009.11.006

[57] Tang HS, Kwan CT, He J et al (2023) Prognostic utility of cardiac MRI myocardial strain parameters in patients with ischemic and nonischemic dilated cardiomyopathy: a multicenter study. AJR Am J Roentgenol 220:524–538 36321987 10.2214/AJR.22.28415

[58] Lu JC, Connelly JA, Zhao L et al (2014) Strain measurement by cardiovascular magnetic resonance in pediatric cancer survivors: validation of feature tracking against harmonic phase imaging. Pediatr Radiol 44:1070–1076 24760125 10.1007/s00247-014-2992-2

[59] Osman NF, Sampath S, Atalar E, Prince JL (2001) Imaging longitudinal cardiac strain on short-axis images using strain-encoded MRI. Magn Reson Med 46:324–334 11477637 10.1002/mrm.1195

[60] Pan L, Stuber M, Kraitchman DL et al (2006) Real-time imaging of regional myocardial function using fast-SENC. Magn Reson Med 55:386–395 16402379 10.1002/mrm.20770

[61] Giusca S, Korosoglou G, Zieschang V et al (2018) Reproducibility study on myocardial strain assessment using fast-SENC cardiac magnetic resonance imaging. Sci Rep 8:14100 30237411 10.1038/s41598-018-32226-3 PMC6147889

[62] Korosoglou G, Youssef AA, Bilchick KC et al (2008) Real-time fast strain-encoded magnetic resonance imaging to evaluate regional myocardial function at 3.0 Tesla: comparison to conventional tagging. J Magn Reson Imaging 27:1012–1018 18407541 10.1002/jmri.21315

[63] Giusca S, Korosoglou G, Montenbruck M et al (2021) Multiparametric early detection and prediction of cardiotoxicity using myocardial strain, T1 and T2 mapping, and biochemical markers: a longitudinal cardiac resonance imaging study during 2 years of follow-up. Circ Cardiovasc Imaging 14:e012459 34126756 10.1161/CIRCIMAGING.121.012459 PMC8208092

[64] Korosoglou G, Giusca S, Montenbruck M et al (2021) Fast strain-encoded cardiac magnetic resonance for diagnostic classification and risk stratification of heart failure patients. JACC Cardiovasc Imaging 14:1177–1188 33454266 10.1016/j.jcmg.2020.10.024

[65] Steen H, Montenbruck M, Gerzak B et al (2020) Intramyocardial fast-SENC CMR strain is less impacted by compensatory mechanisms than echocardiography in monitoring cardiotoxicity: The Prefect Study. J Am College Cardiol 75:1795–1795

[66] Aletras AH, Ding S, Balaban RS, Wen H (1999) DENSE: displacement encoding with stimulated echoes in cardiac functional MRI. J Magn Reson 137:247–252 10053155 10.1006/jmre.1998.1676 PMC2887318

[67] Kim D, Gilson WD, Kramer CM, Epstein FH (2004) Myocardial tissue tracking with two-dimensional cine displacement-encoded MR imaging: development and initial evaluation. Radiology 230:862–871 14739307 10.1148/radiol.2303021213

[68] Spottiswoode BS, Zhong X, Hess AT et al (2007) Tracking myocardial motion from cine DENSE images using spatiotemporal phase unwrapping and temporal fitting. IEEE Trans Med Imaging 26:15–30 17243581 10.1109/TMI.2006.884215

[69] Young AA, Li B, Kirton RS, Cowan BR (2012) Generalized spatiotemporal myocardial strain analysis for DENSE and SPAMM imaging. Magn Reson Med 67:1590–1599 22135133 10.1002/mrm.23142

[70] Auger DA, Ghadimi S, Cai X et al (2022) Reproducibility of global and segmental myocardial strain using cine DENSE at 3 T: a multicenter cardiovascular magnetic resonance study in healthy subjects and patients with heart disease. J Cardiovasc Magn Reson 24:23 35369885 10.1186/s12968-022-00851-7 PMC8978361

[71] Lin K, Meng L, Collins JD et al (2017) Reproducibility of cine displacement encoding with stimulated echoes (DENSE) in human subjects. Magn Reson Imaging 35:148–153 27569367 10.1016/j.mri.2016.08.009 PMC5125889

[72] Kar J, Cohen MV, McQuiston SA et al (2019) Can post-chemotherapy cardiotoxicity be detected in long-term survivors of breast cancer via comprehensive 3D left-ventricular contractility (strain) analysis? Magn Reson Imaging 62:94–103 31254595 10.1016/j.mri.2019.06.020 PMC7170178

[73] Ernande L, Thibault H, Bergerot C et al (2012) Systolic myocardial dysfunction in patients with type 2 diabetes mellitus: identification at MR imaging with cine displacement encoding with stimulated echoes. Radiology 265:402–409 22929334 10.1148/radiol.12112571 PMC3480812

[74] Dahiya A, Chao C, Younger J et al (2021) Society for Cardiovascular Magnetic Resonance 2019 Case of the Week series. J Cardiovasc Magn Reson 23:44 33794918 10.1186/s12968-020-00671-7 PMC8015162

[75] Chen X, Yang Y, Cai X et al (2016) Accelerated two-dimensional cine DENSE cardiovascular magnetic resonance using compressed sensing and parallel imaging. J Cardiovasc Magn Reson 18:38 27301487 10.1186/s12968-016-0253-2 PMC4906684

[76] Ghadimi S, Auger DA, Feng X et al (2021) Fully-automated global and segmental strain analysis of DENSE cardiovascular magnetic resonance using deep learning for segmentation and phase unwrapping. J Cardiovasc Magn Reson 23:20 33691739 10.1186/s12968-021-00712-9 PMC7949250

[77] Schuster A, Hor KN, Kowallick JT et al (2016) Cardiovascular magnetic resonance myocardial feature tracking: concepts and clinical applications. Circ Cardiovasc Imaging 9:e004077 27009468 10.1161/CIRCIMAGING.115.004077

[78] Korosoglou G, Sagris M, Andre F et al (2024) Systematic review and meta-analysis for the value of cardiac magnetic resonance strain to predict cardiac outcomes. Sci Rep 14:1094 38212323 10.1038/s41598-023-50835-5 PMC10784294

[79] Lee BH, Goodenday LS, Muswick GJ et al (1987) Alterations in left ventricular diastolic function with doxorubicin therapy. J Am Coll Cardiol 9:184–188 3794095 10.1016/s0735-1097(87)80099-2

[80] Marchandise B, Schroeder E, Bosly A et al (1989) Early detection of doxorubicin cardiotoxicity: interest of Doppler echocardiographic analysis of left ventricular filling dynamics. Am Heart J 118:92–98 2741800 10.1016/0002-8703(89)90077-x

[81] Stoddard MF, Seeger J, Liddell NE et al (1992) Prolongation of isovolumetric relaxation time as assessed by Doppler echocardiography predicts doxorubicin-induced systolic dysfunction in humans. J Am Coll Cardiol 20:62–69 1607540 10.1016/0735-1097(92)90138-d

[82] Honda K, Takeshita K, Murotani K et al (2017) Assessment of left ventricular diastolic function during trastuzumab treatment in patients with HER2-positive breast cancer. Breast Cancer 24:312–318 27234030 10.1007/s12282-016-0705-4

[83] Klein R, Nadouri D, Osler E et al (2019) Diastolic dysfunction can precede systolic dysfunction on MUGA in cancer patients receiving trastuzumab-based therapy. Nucl Med Commun 40:22–29 30418380 10.1097/MNM.0000000000000941 PMC6282666

[84] Upshaw JN, Finkelman B, Hubbard RA et al (2020) Comprehensive assessment of changes in left ventricular diastolic function with contemporary breast cancer therapy. JACC Cardiovasc Imaging 13:198–210 31542526 10.1016/j.jcmg.2019.07.018 PMC7236624

[85] Sulicka-Grodzicka J, Chyrchel B, Toton-Zuranska J et al (2019) Cranial irradiation in childhood acute lymphoblastic leukemia is related to subclinical left ventricular dysfunction and reduced large artery compliance in cancer survivors. J Clin Med 8:1952 31766118 10.3390/jcm8111952 PMC6912438

[86] Palmer C, Mazur W, Truong VT et al (2023) Prevalence of diastolic dysfunction in adult survivors of childhood cancer: a report from SJLIFE Cohort. JACC CardioOncol 5:377–388 37397075 10.1016/j.jaccao.2022.12.010 PMC10308058

[87] Paelinck BP, Lamb HJ, Bax JJ et al (2002) Assessment of diastolic function by cardiovascular magnetic resonance. Am Heart J 144:198–205 12177633 10.1067/mhj.2002.123316

[88] Mohiaddin RH, Amanuma M, Kilner PJ et al (1991) MR phase-shift velocity mapping of mitral and pulmonary venous flow. J Comput Assist Tomogr 15:237–243 2002101 10.1097/00004728-199103000-00009

[89] Farhad H, Seidelmann SB, Vigneault D et al (2017) Left atrial structure and function in hypertrophic cardiomyopathy sarcomere mutation carriers with and without left ventricular hypertrophy. J Cardiovasc Magn Reson 19:107 29284499 10.1186/s12968-017-0420-0 PMC5745877

[90] Dodson JA, Neilan TG, Shah RV et al (2014) Left atrial passive emptying function determined by cardiac magnetic resonance predicts atrial fibrillation recurrence after pulmonary vein isolation. Circ Cardiovasc Imaging 7:586–592 24902586 10.1161/CIRCIMAGING.113.001472 PMC4219259

[91] Kikano SD, Weingarten A, Sunthankar SD et al (2023) Association of cardiovascular magnetic resonance diastolic indices with arrhythmia in repaired Tetralogy of Fallot. J Cardiovasc Magn Reson 25:17 36907898 10.1186/s12968-023-00928-x PMC10009941

[92] Singh A, Steadman CD, Khan JN et al (2015) Intertechnique agreement and interstudy reproducibility of strain and diastolic strain rate at 1.5 and 3 Tesla: a comparison of feature-tracking and tagging in patients with aortic stenosis. J Magn Reson Imaging 41:1129–1137 24700404 10.1002/jmri.24625

[93] Hinojar R, Zamorano JL, Fernandez-Mendez M et al (2019) Prognostic value of left atrial function by cardiovascular magnetic resonance feature tracking in hypertrophic cardiomyopathy. Int J Cardiovasc Imaging 35:1055–1065 30706353 10.1007/s10554-019-01534-8

[94] Barbosa MF, Fusco DR, Gaiolla RD et al (2021) Characterization of subclinical diastolic dysfunction by cardiac magnetic resonance feature-tracking in adult survivors of non-Hodgkin lymphoma treated with anthracyclines. BMC Cardiovasc Disord 21:170 33845778 10.1186/s12872-021-01996-6 PMC8040217

[95] van Schinkel LD, Willemse PM, van der Meer RW et al (2013) Chemotherapy for testicular cancer induces acute alterations in diastolic heart function. Br J Cancer 109:891–896 23922115 10.1038/bjc.2013.445 PMC3749589

[96] Song L, Brezden-Masley C, Ramanan V et al (2019) Serial measurements of left ventricular systolic and diastolic function by cardiac magnetic resonance imaging in patients with early stage breast cancer on trastuzumab. Am J Cardiol 123:1173–1179 30683420 10.1016/j.amjcard.2018.12.046

[97] O’Quinn R, Ferrari VA, Daly R et al (2021) Cardiac magnetic resonance in cardio-oncology: advantages, importance of expediency, and considerations to navigate pre-authorization. JACC CardioOncol 3:191–200 34396324 10.1016/j.jaccao.2021.04.011 PMC8352183

[98] Anghel N, Herman H, Balta C et al (2018) Acute cardiotoxicity induced by doxorubicin in right ventricle is associated with increase of oxidative stress and apoptosis in rats. Histol Histopathol 33:365–378 28920632 10.14670/HH-11-932

[99] Campian ME, Verberne HJ, Hardziyenka M et al (2009) Serial noninvasive assessment of apoptosis during right ventricular disease progression in rats. J Nucl Med 50:1371–1377 19617336 10.2967/jnumed.108.061366

[100] Cardinale D, Sandri MT, Martinoni A et al (2000) Left ventricular dysfunction predicted by early troponin I release after high-dose chemotherapy. J Am Coll Cardiol 36:517–522 10933366 10.1016/s0735-1097(00)00748-8

[101] Rossetto L, Di Lisi D, Madaudo C et al (2024) Right ventricle involvement in patients with breast cancer treated with chemotherapy. Cardiooncology 10:24 38616279 10.1186/s40959-024-00224-2 PMC11017635

[102] Shen Y, Zhang H, Zhang Q et al (2022) Right ventricular ejection fraction assessed by three-dimensional echocardiography is associated with long-term adverse clinical cardiac events in patients with anthracycline-induced cardiotoxicity. J Am Soc Echocardiogr 35(600–608):e603 10.1016/j.echo.2022.01.018 35158050

[103] Messroghli DR, Moon JC, Ferreira VM et al (2017) Clinical recommendations for cardiovascular magnetic resonance mapping of T1, T2, T2* and extracellular volume: a consensus statement by the Society for Cardiovascular Magnetic Resonance (SCMR) endorsed by the European Association for Cardiovascular Imaging (EACVI). J Cardiovasc Magn Reson 19:75 28992817 10.1186/s12968-017-0389-8 PMC5633041

[104] Toro-Salazar OH, Gillan E, O’Loughlin M et al (2013) Occult cardiotoxicity in childhood cancer survivors exposed to anthracycline therapy. Circ Cardiovasc Imaging 6:873–80 24097420 10.1161/CIRCIMAGING.113.000798

[105] Lee E, Ibrahim EH, Parwani P et al (2020) Practical guide to evaluating myocardial disease by cardiac MRI. AJR Am J Roentgenol 214:546–556 31967503 10.2214/AJR.19.22076

[106] Pagano JJ, Yim D, Lam CZ et al (2020) Normative data for myocardial native T1 and extracellular volume fraction in children. Radiol Cardiothorac Imaging 2:e190234 33778602 10.1148/ryct.2020190234 PMC7977704

[107] Hanson CA, Kamath A, Gottbrecht M et al (2020) T2 Relaxation times at cardiac MRI in healthy adults: a systematic review and meta-analysis. Radiology 297:344–351 32840469 10.1148/radiol.2020200989 PMC7605362

[108] Moon JC, Messroghli DR, Kellman P et al (2013) Myocardial T1 mapping and extracellular volume quantification: a Society for Cardiovascular Magnetic Resonance (SCMR) and CMR Working Group of the European Society of Cardiology consensus statement. J Cardiovasc Magn Reson 15:92 24124732 10.1186/1532-429X-15-92 PMC3854458

[109] Hundley WG, Jordan JH (2018) When left ventricular extracellular volume fraction changes after anthracyclines: is it due to a change in the numerator, denominator, or both? JACC Cardiovasc Imaging 11:1056–1058 30092966 10.1016/j.jcmg.2018.06.006 PMC8565717

[110] Gannon MP, Schaub E, Grines CL, Saba SG (2019) State of the art: evaluation and prognostication of myocarditis using cardiac MRI. J Magn Reson Imaging 49:e122–e131 30637834 10.1002/jmri.26611

[111] Tham EB, Haykowsky MJ, Chow K et al (2013) Diffuse myocardial fibrosis by T1-mapping in children with subclinical anthracycline cardiotoxicity: relationship to exercise capacity, cumulative dose and remodeling. J Cardiovasc Magn Reson 15:48 23758789 10.1186/1532-429X-15-48 PMC3688348

[112] Mawad W, Mertens L, Pagano JJ et al (2021) Effect of anthracycline therapy on myocardial function and markers of fibrotic remodelling in childhood cancer survivors. Eur Heart J Cardiovasc Imaging 22:435–442 32535624 10.1093/ehjci/jeaa093 PMC7984732

[113] Neilan TG, Coelho-Filho OR, Shah RV et al (2013) Myocardial extracellular volume by cardiac magnetic resonance imaging in patients treated with anthracycline-based chemotherapy. Am J Cardiol 111:717–722 23228924 10.1016/j.amjcard.2012.11.022 PMC3578020

[114] Folco G, Monti CB, Zanardo M et al (2024) MRI-derived extracellular volume as a biomarker of cancer therapy cardiotoxicity: systematic review and meta-analysis. Eur Radiol 34:2699–2710 37823922 10.1007/s00330-023-10260-8 PMC10957707

[115] Ferreira VM, Schulz-Menger J, Holmvang G et al (2018) Cardiovascular magnetic resonance in nonischemic myocardial inflammation: expert recommendations. J Am Coll Cardiol 72:3158–3176 30545455 10.1016/j.jacc.2018.09.072

[116] Greulich S, Ferreira VM, Dall’Armellina E, Mahrholdt H (2015) Myocardial inflammation-are we there yet? Curr Cardiovasc Imaging Rep 8:6 25705323 10.1007/s12410-015-9320-6 PMC4330458

[117] Galán-Arriola C, Lobo M, Vílchez-Tschischke JP et al (2019) Serial magnetic resonance imaging to identify early stages of anthracycline-induced cardiotoxicity. J Am Coll Cardiol 73:779–791 30784671 10.1016/j.jacc.2018.11.046

[118] Thavendiranathan P, Shalmon T, Fan CS et al (2023) Comprehensive cardiovascular magnetic resonance tissue characterization and cardiotoxicity in women with breast cancer. JAMA Cardiol 8:524–534 37043251 10.1001/jamacardio.2023.0494 PMC10099158

[119] Zhao SH, Yun H, Chen CZ et al (2022) Applying quantitative CMR parameters for detecting myocardial lesion in immune checkpoint inhibitors-associated myocarditis. Eur J Radiol 156:110558 36265221 10.1016/j.ejrad.2022.110558

[120] Schempp A, Lee J, Kearney S et al (2016) Iron overload in survivors of childhood cancer. J Pediatr Hematol Oncol 38:27–31 26422286 10.1097/MPH.0000000000000444

[121] Ruccione KS, Wood JC, Sposto R et al (2014) Characterization of transfusion-derived iron deposition in childhood cancer survivors. Cancer Epidemiol Biomarkers Prev 23:1913–1919 24962841 10.1158/1055-9965.EPI-14-0292

[122] Lambing A, Kachalsky E, Mueller ML (2012) The dangers of iron overload: bring in the iron police. J Am Acad Nurse Pract 24:175–183 22486832 10.1111/j.1745-7599.2011.00680.x

[123] Bardi E, Mulder RL, van Dalen EC et al (2021) Late hepatic toxicity surveillance for survivors of childhood, adolescent and young adult cancer: recommendations from the international late effects of childhood cancer guideline harmonization group. Cancer Treat Rev 100:102296 34571378 10.1016/j.ctrv.2021.102296

[124] de Ville de Goyet M, Moniotte S, Robert A et al (2013) Iron overload in children undergoing cancer treatments. Pediatr Blood Cancer 60:1982-1987 10.1002/pbc.24705 23897631

[125] Jenista ER, Wendell DC, Azevedo CF et al (2023) Revisiting how we perform late gadolinium enhancement CMR: insights gleaned over 25 years of clinical practice. J Cardiovasc Magn Reson 25:18 36922844 10.1186/s12968-023-00925-0 PMC10018965

[126] Kim RJ, Fieno DS, Parrish TB et al (1999) Relationship of MRI delayed contrast enhancement to irreversible injury, infarct age, and contractile function. Circulation 100:1992–2002 10556226 10.1161/01.cir.100.19.1992

[127] Flood TF, Stence NV, Maloney JA, Mirsky DM (2017) Pediatric brain: repeated exposure to linear gadolinium-based contrast material is associated with increased signal intensity at unenhanced T1-weighted MR Imaging. Radiology 282:222–228 27467467 10.1148/radiol.2016160356

[128] Yancy CW, Jessup M, Bozkurt B et al (2013) 2013 ACCF/AHA guideline for the management of heart failure: executive summary: a report of the American College of Cardiology Foundation/American Heart Association Task Force on practice guidelines. Circulation 128:1810–1852 23741057 10.1161/CIR.0b013e31829e8807

[129] Lightfoot JC, D’Agostino RB Jr, Hamilton CA et al (2010) Novel approach to early detection of doxorubicin cardiotoxicity by gadolinium-enhanced cardiovascular magnetic resonance imaging in an experimental model. Circ Cardiovasc Imaging 3:550–558 20622140 10.1161/CIRCIMAGING.109.918540 PMC3068484

[130] Steinherz LJ, Steinherz PG, Tan CT et al (1991) Cardiac toxicity 4 to 20 years after completing anthracycline therapy. JAMA 266:1672–1677 1886191

[131] Jordan JH, D’Agostino RB Jr, Hamilton CA et al (2014) Longitudinal assessment of concurrent changes in left ventricular ejection fraction and left ventricular myocardial tissue characteristics after administration of cardiotoxic chemotherapies using T1-weighted and T2-weighted cardiovascular magnetic resonance. Circ Cardiovasc Imaging 7:872–879 25273568 10.1161/CIRCIMAGING.114.002217 PMC4241241

[132] Harries I, Biglino G, Baritussio A et al (2019) Long term cardiovascular magnetic resonance phenotyping of anthracycline cardiomyopathy. Int J Cardiol 292:248–252 31006597 10.1016/j.ijcard.2019.04.026

[133] Modi K, Joppa S, Chen KA et al (2021) Myocardial damage assessed by late gadolinium enhancement on cardiovascular magnetic resonance imaging in cancer patients treated with anthracyclines and/or trastuzumab. Eur Heart Cardiovasc Imaging 22:427–434 10.1093/ehjci/jeaa279 PMC7984730 33211843

[134] Ylänen K, Poutanen T, Savikurki-Heikkilä P et al (2013) Cardiac magnetic resonance imaging in the evaluation of the late effects of anthracyclines among long-term survivors of childhood cancer. J Am College Cardiol 61:1539–1547 10.1016/j.jacc.2013.01.019 23500246

[135] Wolf CM, Reiner B, Kühn A et al (2020) Subclinical cardiac dysfunction in childhood cancer survivors on 10-years follow-up correlates with cumulative anthracycline dose and is best detected by cardiopulmonary exercise testing, circulating serum biomarker, speckle tracking echocardiography, and tissue Doppler imaging. Front Pediatr 8:123 32296665 10.3389/fped.2020.00123 PMC7136405

[136] Tong X, Li VW, Liu AP et al (2019) Cardiac magnetic resonance T1 mapping in adolescent and young adult survivors of childhood cancers. Circ Cardiovasc Imaging 12:e008453 30929466 10.1161/CIRCIMAGING.118.008453

[137] Long TM, Marsh CE, Dembo LG et al (2019) Early markers of cardiovascular injury in childhood leukaemia survivors treated with anthracycline chemotherapy. Cardio-oncology (London, England) 5:11 32154017 10.1186/s40959-019-0047-4 PMC7048057

[138] Cheung YF, Lam WW, Ip JJ et al (2015) Myocardial iron load and fibrosis in long term survivors of childhood leukemia. Pediatr Blood Cancer 62:698–703 25557466 10.1002/pbc.25369

[139] Kim HW, Rehwald WG, Jenista ER et al (2018) Dark-blood delayed enhancement cardiac magnetic resonance of myocardial infarction. JACC Cardiovasc Imaging 11:1758–1769 29248655 10.1016/j.jcmg.2017.09.021 PMC5993564

[140] Bissell MM, Raimondi F, Ait Ali L et al (2023) 4D Flow cardiovascular magnetic resonance consensus statement: 2023 update. J Cardiovasc Magn Reson 25:40 37474977 10.1186/s12968-023-00942-z PMC10357639

[141] Ashkir Z, Myerson S, Neubauer S et al (2022) Four-dimensional flow cardiac magnetic resonance assessment of left ventricular diastolic function. Front Cardiovasc Med 9:866131 35935619 10.3389/fcvm.2022.866131 PMC9355735

[142] Shen H, Zhou W, ChunrongTu, et al (2024) Thoracic aorta injury detected by 4D flow MRI predicts subsequent main adverse cardiovascular events in breast cancer patients receiving anthracyclines: a longitudinal study. Magn Reson Imaging 109:67–73 38484947 10.1016/j.mri.2024.03.010

[143] Alattar Y, Soulat G, Gencer U et al (2022) Left ventricular diastolic early and late filling quantified from 4D flow magnetic resonance imaging. Diagn Interv Imaging 103:345–352 35227634 10.1016/j.diii.2022.02.003

[144] Sakakibara T, Suwa K, Ushio T et al (2021) Intra-left ventricular hemodynamics assessed with 4D flow magnetic resonance imaging in patients with left ventricular thrombus. Int Heart J 62:1287–1296 34853222 10.1536/ihj.20-792

[145] Eriksson J, Bolger AF, Ebbers T, Carlhall CJ (2016) Assessment of left ventricular hemodynamic forces in healthy subjects and patients with dilated cardiomyopathy using 4D flow MRI. Physiol Rep 4:e12685 26841965 10.14814/phy2.12685 PMC4758930

[146] Arvidsson PM, Toger J, Pedrizzetti G et al (2018) Hemodynamic forces using four-dimensional flow MRI: an independent biomarker of cardiac function in heart failure with left ventricular dyssynchrony? Am J Physiol Heart Circ Physiol 315:H1627–H1639 30216113 10.1152/ajpheart.00112.2018

[147] Pourier M, Merkx R, Loonen J et al (2022) Cardiac events in childhood cancer survivors treated with anthracyclines: the value of previous myocardial strain measurement. Life (Basel) 12:452 35330203 10.3390/life12030452 PMC8953171

[148] Plana JC, Galderisi M, Barac A et al (2014) Expert consensus for multimodality imaging evaluation of adult patients during and after cancer therapy: a report from the American Society of Echocardiography and the European Association of Cardiovascular Imaging. J Am Soc Echocardiogr 27:911–939 25172399 10.1016/j.echo.2014.07.012

[149] Curigliano G, Cardinale D, Suter T et al (2012) Cardiovascular toxicity induced by chemotherapy, targeted agents and radiotherapy: ESMO Clinical Practice Guidelines. Ann Oncol 23(Suppl 7):155–166 10.1093/annonc/mds293 22997448

[150] Dixon SB, Howell CR, Lu L et al (2021) Cardiac biomarkers and association with subsequent cardiomyopathy and mortality among adult survivors of childhood cancer: a report from the St. Jude Lifetime Cohort. Cancer 127:458–466 33108003 10.1002/cncr.33292 PMC7855049

[151] Ehrhardt MJ, Liu Q, Mulrooney DA et al (2024) Improved cardiomyopathy risk prediction using global longitudinal strain and N-terminal-pro-B-type natriuretic peptide in survivors of childhood cancer exposed to cardiotoxic therapy. J Clin Oncol 42:1265–1277 38207238 10.1200/JCO.23.01796 PMC11095874

[152] Winther HB, Hundt C, Schmidt B, Czerner C, Bauersachs J, Wacker F, Vogel-Claussen J (2018) V-net: deep learning for generalized biventricular mass and function parameters using multicenter cardiac MRI data. JACC Cardiovasc Imaging 11:1036–1038 29361481 10.1016/j.jcmg.2017.11.013

[153] Tan LK, Liew YM, Lim E, McLaughlin RA (2017) Convolutional neural network regression for short-axis left ventricle segmentation in cardiac cine MR sequences. Med Image Anal 39:78–86 28437634 10.1016/j.media.2017.04.002

[154] Bai W, Sinclair M, Tarroni G et al (2018) Automated cardiovascular magnetic resonance image analysis with fully convolutional networks. J Cardiovasc Magn Reson 20:65 30217194 10.1186/s12968-018-0471-x PMC6138894

[155] Hamilton JI, Jiang Y, Chen Y et al (2017) MR fingerprinting for rapid quantification of myocardial T(1), T(2), and proton spin density. Magn Reson Med 77:1446–1458 27038043 10.1002/mrm.26216 PMC5045735

[156] Liu Y, Hamilton J, Rajagopalan S, Seiberlich N (2018) Cardiac magnetic resonance fingerprinting: technical overview and initial results. JACC Cardiovasc Imaging 11:1837–1853 30522686 10.1016/j.jcmg.2018.08.028 PMC6394856

[157] Kwan JM, Arbune A, Henry ML et al (2023) Quantitative cardiovascular magnetic resonance findings and clinical risk factors predict cardiovascular outcomes in breast cancer patients. PLoS One 18:e0286364 37252927 10.1371/journal.pone.0286364 PMC10228774

[158] Craven TP, Tsao CW, La Gerche A et al (2020) Exercise cardiovascular magnetic resonance: development, current utility and future applications. J Cardiovasc Magn Reson 22:65 32907587 10.1186/s12968-020-00652-w PMC7488086

[159] Friedrich MG, Karamitsos TD (2013) Oxygenation-sensitive cardiovascular magnetic resonance. J Cardiovasc Magn Reson 15:43 23706167 10.1186/1532-429X-15-43 PMC3681671

[160] DiLorenzo MP, Lee S, Rathod RH et al (2024) Design and implementation of multicenter pediatric and congenital studies with cardiovascular magnetic resonance: big data in smaller bodies. J Cardiovasc Magn Reson 26:101041 38527706 10.1016/j.jocmr.2024.101041 PMC10990896

